# A subtractive proteomics approach for the identification of immunodominant *Acinetobacter baumannii* vaccine candidate proteins

**DOI:** 10.3389/fimmu.2022.1001633

**Published:** 2022-11-10

**Authors:** Mustafa Burak Acar, Şerife Ayaz-Güner, Hüseyin Güner, Gökçen Dinç, Ayşegül Ulu Kılıç, Mehmet Doğanay, Servet Özcan

**Affiliations:** ^1^ Department of Biology, Faculty of Science, Erciyes University, Kayseri, Turkey; ^2^ Genome and Stem Cell Center (GENKÖK), Erciyes University, Kayseri, Turkey; ^3^ Department of Molecular Biology and Genetics, Faculty of Life and Natural Science, Abdullah Gül University, Kayseri, Turkey; ^4^ Department of Molecular Biology and Genetics, Izmir Institute of Technology, Izmir, Turkey; ^5^ Department of Medical Microbiology, Faculty of Medicine, Erciyes University, Kayseri, Turkey; ^6^ Department of Infectious Disease and Clinical Microbiology, Faculty of Medicine, Erciyes University, Kayseri, Turkey; ^7^ Department of Infectious Diseases, Faculty of Medicine, Lokman Hekim University, Ankara, Turkey

**Keywords:** *Acinetobacter baumannii* (A. baumannii), vaccine candidate, proteomics, immunoprecipitation, immunodominant

## Abstract

**Background:**

*Acinetobacter baumannii* is one of the most life-threatening multidrug-resistant pathogens worldwide. Currently, 50%–70% of clinical isolates of *A. baumannii* are extensively drug-resistant, and available antibiotic options against *A. baumannii* infections are limited. There is still a need to discover specific *de facto* bacterial antigenic proteins that could be effective vaccine candidates in human infection. With the growth of research in recent years, several candidate molecules have been identified for vaccine development. So far, no public health authorities have approved vaccines against *A. baumannii*.

**Methods:**

This study aimed to identify immunodominant vaccine candidate proteins that can be immunoprecipitated specifically with patients’ IgGs, relying on the hypothesis that the infected person’s IgGs can capture immunodominant bacterial proteins. Herein, the outer-membrane and secreted proteins of sensitive and drug-resistant *A. baumannii* were captured using IgGs obtained from patient and healthy control sera and identified by Liquid Chromatography- Tandem Mass Spectrometry (LC-MS/MS) analysis.

**Results:**

Using the subtractive proteomic approach, we determined 34 unique proteins captured only in drug-resistant *A. baumannii* strain *via* patient sera. After extensively evaluating the predicted epitope regions, solubility, transverse membrane characteristics, and structural properties, we selected several notable vaccine candidates.

**Conclusion:**

We identified vaccine candidate proteins that triggered a *de facto* response of the human immune system against the antibiotic-resistant *A. baumannii*. Precipitation of bacterial proteins *via* patient immunoglobulins was a novel approach to identifying the proteins that could trigger a response in the patient immune system.

## Graphical Abstract

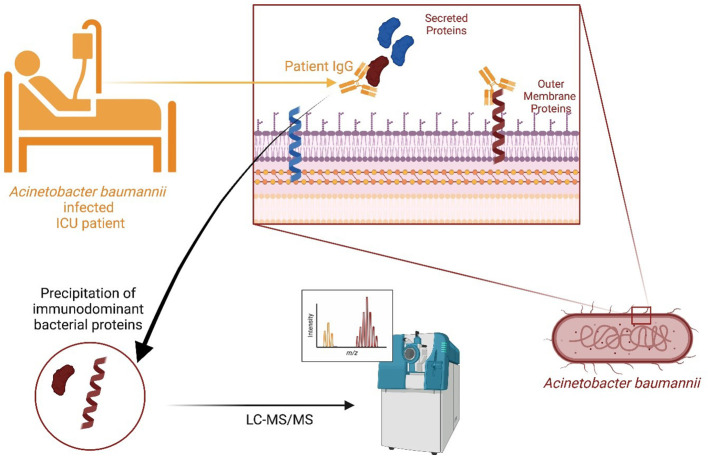

## Introduction

*Acinetobacter* spp. are gram-negative, aerobic, non-fermentative, stabile, and bacillus-shaped bacteria that reside in sand and water. Approximately 25% of healthy people can contain these bacteria in their armpit, groin, and even the oral cavity and respiratory tract (1, 2). These species form the pinky colonies on Mac Conkey agar; can be shown as bacillus, coccobacillus; and can survive even in dry conditions due to biofilm formation (3, 4).

An intravascular, ventricular catheter or endotracheal tube insertion can increase the risk of nosocomial infection following surgical operations. *A. baumannii* can surround the wound site or damaged mucosal areas. Being an opportunistic bacterium, *A. baumannii* mainly infects critically ill immunocompromised patients in intensive care units (ICUs) (5). Despite the treatment with combined antibiotics, emerging multidrug resistance makes the treatment ineffective, and infection caused by these bacteria can result in death (6).

*A. baumannii* is one of the most life-threatening multidrug-resistant pathogens globally. Currently, 50%–70% of *A. baumannii* clinical isolates have extensive drug resistance, and the frequency of the infections caused by these bacteria is increasing (7, 8). In developing resistance against colistin and tigecycline, *A. baumannii* may become a pan-drug-resistant bacterium that causes an infection incurable by Food and Drug Administration (FDA)-approved antibiotics (9, 10).

There is an ongoing need for an effective vaccine candidate that protects against *A. baumannii* infection. Running out of antibiotic options available against *A. baumannii* reveals the necessity of vaccination and the development of alternative treatment approaches against this bacterium. Several vaccine candidate molecules have been identified by the increasing number of studies in recent years. The antigens used in these studies include outer-membrane proteins (OMPs) such as OmpA, Omp34, and OprC Phospholipase C and D, which play an essential role in the virulence and biofilm genesis of this bacterium (6, 11–15). In particular, -omics and *in silico* techniques with the potential of generating and analyzing the high-throughput data identified vaccine candidate molecules. Unfortunately, unlike other opportunistic bacterial infections, there is no available vaccine approved by health authorities for *A. baumannii* (16).

The present study aimed to identify those potential vaccine candidate proteins that can be specifically immunoprecipitated with the IgGs that present only in patients suffering from *A. baumannii* infections and non-responsive to antibiotic treatment. We excluded bacterial antigens immunoprecipitated by control sera to determine patient-specific ones. Therefore, the outer-membrane and secreted proteins of sensitive and drug-resistant bacteria were captured using immunoglobulin-containing patient and control sera and identified by LC-MS/MS analysis. Our approach allowed us to identify 34 unique bacterial proteins that triggered an immune response in infected individuals but not in the control and healthy individuals.

By performing bioinformatic evaluations, epitope prediction, solubility, transmembrane properties, and structural specifications, nine selected proteins were further evaluated for possible recombinant applications. Hence, several vaccine candidates that could potentially protect against the drug-resistant *A. baumannii* were identified.

## Material and methods

### Blood sample collection

Blood samples *of A. baumannii* bacteremia–diagnosed patients, kept in the ICU of the Erciyes University Hospital, were collected with the approval of the local Human Ethical Committees (ERU LEC_2013-445) and with the written consent of the patients. Twenty-nine infection-positive cases, 13 non-infected patients as the negative control of the ICU, and three as the external control group from healthy individuals were used for this study. Demographic features of the patients are summarized in **Supplementary Table 1**. Whole blood (10 ml) was collected into Vacutainer sample tubes and centrifuged at 3,000 rpm for 10 min. Serum samples were stored at −80°C for further analysis.

### Bacterial strain, growth conditions, and protein sample collection

Multiple drug-resistant (BAA-1710) and non-resistant (ATCC 17978) *A. baumannii* strains were purchased from American Type Culture Collection (ATCC). *A. baumannii* ATCC^®^ BAA-1710™ is resistant to cefazolin, cefepime, cefotaxime, ceftazidime, ceftriaxone, ciprofloxacin, gentamicin, levofloxacin, piperacillin, piperacillin-tazobactam, ticarcillin, tetracycline, and trimethoprim-sulfamethoxazole, and this strain is susceptible to ampicillin-sulbactam, doripenem, imipenem, meropenem, and tigecycline (https://www.atcc.org/products/baa-1710#detailed-product-information). *A. baumannii* ATCC^®^ 17978™ is susceptible strain that is preferred as reference strain in similar studies (https://www.atcc.org/products/17978) (17, 18).

Well-isolated colonies of both strains were grown in Luria-Bertani broth overnight at 37°C and 200 rpm on an orbital shaker. 1:100 (v/v) dilution of the culture was started and grown until OD_600_ reached 1.0-1.2. Suspension of bacteria was chilled on ice and centrifuged at 4°C and 8,000 x *g* for 20 min. Supernatants were used for secretome analysis to perform immunoproteomic analysis, and the pellets were subjected to OMP isolation.

### Naïve secretome protein isolation

Secreted protein was harvested as mentioned before, and to increase the analysis coverage and obtain the secreted proteins in their natural confirmation, conditional medium was directly used for immunoprecipitation. MultiMACS protein A/G microbeads (Miltenyi Biotec, Germany) were used to catch the secreted protein described by (19).

### Protein isolation by phenol extraction method

Conditional media of *A. baumannii* were collected, and 1:8 (v/v) phenol (Sigma-Aldrich, Germany) was added. After 30 min of incubation at 4°C, samples were centrifuged at 6,000 x *g* for 20 min. Protein-containing phenol phase was transferred to a new collection tube. Equal volume of Back Extraction Buffer [0.1 M Tris-HCl (pH 8), 20 mM KCl, 10 mM Ethylenediaminetetraacetic acid (EDTA), and 0.4% β-mercaptoethanol] was added to each sample (20). After 15 min of incubation at room temperature, samples were centrifuged at 6,000 x *g* for 15 min. The previous step was repeated, and the phenol phase was transferred to a new tube. Precipitation buffer (0.1 M NH_4_OAc in methanol) was added to the sample at a 5:1 (v/v) ratio, and samples were incubated at −20°C overnight. After centrifugation at 4°C and 15,000 x *g* for 30 min, the pellet was washed twice with 80% acetone and dried with SpeedVac (Eppendorf, USA) at 30°C for 1 h. Finally, phenol-precipitated proteins were resuspended with 500 μl of Cell Lysis Buffer (CLB) [150 mM NaCl, 50 mM Tris-HCl (pH 8), and 0.1% Triton X-100] followed by immunoprecipitation.

### Outer-membrane protein isolation

Bacterial pellets were obtained by centrifugation at 4°C and 5000 *x g* for 10 min, washed twice with Dulbecco's Phosphate Buffered Saline (DPBS), and stored at −80°C overnight. Frozen pellets were thawed and resuspended with buffer (10 mM Tris-HCl, pH 7.5). Protease inhibitors (2%, Roche, without EDTA) were added on suspension and sonicated (Vibra Cell, Sonics, VCX130) for 10 cycles (15-s sonication and 45-s cooldown). The centrifugation process was repeated to get rid of the cytosolic contaminants. The ultracentrifugation (Hitachi, CP100NX) process was performed according to a method described by (21). Samples were centrifuged at 108,000 x *g* and 4°C for 15 min, and 2% Triton X-100 containing 4 ml of Tris-HCl solution was added to the pellet and incubated at room temperature. After incubation, samples were centrifuged with the parameters indicated in the previous step. Membrane proteins containing pellets were dissolved in 500 μl of CLB for immunoprecipitation.

### Immunoprecipitation

To increase the coverage of analysis beyond the limitations of classical immunoblotting methods, the immunoprecipitation approach, which makes it possible to obtain the proteins in their naïve confirmational forms, was used. Enriched proteins (either membrane or secretome) were mixed with patients’ sera and control sera separately and incubated at 4°C for 1 h. To capture IgG-interacting antigens, 50 μl of protein G–conjugated nanobeads (Miltenyi Biotec, Germany) were added to the samples. After 30 min of incubation, the samples were applied on paramagnetic columns that were washed with CLB. Following the passing of samples, columns were washed four times with lysis buffer previously and then washed with a low-salt buffer (20 mM Tris-HCl, pH 7.5) to get rid of the salt and detergent residues. Labeled antigenic molecules were torn off from columns with 100 μl of pre-warmed Laemmli Buffer that contains 50 mM DL-Dithiothreitol (DTT). Eluents were collected for further steps of sample preparation.

### Filter-aided sample preparation of immunoprecipitated proteins

The FASP (filter-aided sample preparation) Protein Digestion Kit (Expedeon, UK) was used in the sample preparation process (22). Following the immunoprecipitation, 30 μl from 100 μl of eluent was mixed with 200 μl of urea sample solution (8 M urea in 100 mM Tris-HCl, pH 8). The protein samples were then transferred to a 30-kDa cutoff spin filter cartridge (FASP kit; Expedeon, Inc.) and centrifuged at room temperature 14,000 x *g* for 15 min. The contaminants were removed by buffer exchange in two successive washes with 200 μl of urea sample solution (8 M urea in 100 mM Tris-HCl, pH 8) with a 15-min centrifugation at 14,000 x *g*. The samples were incubated with iodoacetamide solution for 20 min in the dark and centrifuged at 14,000 x *g* for 10 min. After three successive washes with urea sample solution (8 M urea in 100 mM Tris-HCl, pH 8), the samples were washed with 50 mM ammonium bicarbonate (ABC) solution. Protein digestion was achieved by adding Trypsin/Lys-C (400 ng/ml; Promega, USA) in 50 mM ABC and incubating at 37°C overnight. Following the initial digestion, proteins were digested with Trypsin-Gold (400 ng/ml; Promega, USA) for 6 h at 37°C. Peptides were then eluted with 50 mM ABC solution and 500 mM NaCl sodium chloride (provided in FASP kit; Expedeon, Inc.) by centrifugation at 14,000 x g for 10 min. Collected samples were dried with a vacuum concentrator (Speedvac, Eppendorf, USA).

### ZipTip purification of samples

To remove the salts from samples and purify them, ZipTip C18 (Merck-Millipore, Germany) tips were used in this step. Digested peptides were resolved with 10 μl of 0.1% Trifluoroacetic acid (TFA) (Sigma-Aldrich, Germany) containing mass spectrometry grade water (Fluka, USA). Before sample purification, C18 materials of ZipTips were washed with 10 μl of Acetonitrile (ACN), conditioned twice with 70% ACN and equilibrated twice by using 10 μl of 3% ACN and 0.1% acetic acid. Afterward, resolved samples were pipetted 10 times for each sample by using an equilibrated ZipTip. Following this step, captured peptides in C18 material were washed twice by pipetting of 10 μl of 5% ACN and 0.1% acetic acid, and in this way, impurities were washed out. Purified peptides were eluted from ZipTip using 10 μl of 60% ACN and 0.1% acetic acid and dried using a vacuum concentrator. Before analysis, samples were resolved in 10 μl of 3% ACN and 0.1% formic acid and transferred to thin glass in Mass Spectrometry (MS) vials.

### Mass spectrometry analysis

Shotgun proteomic analyses have been performed with AB SCIEX TripleTOF^®^ 5600+ integrated to Eksigent ekspert™ nanoLC 400 System (AB Sciex, USA). Trap column (3 μm, ChromXP C18CL) and nanoAQUITY UPLC^®^ NanoLC column (1.8-μm HSS T3, 75 μm × 150 mm) were used in the trap-elute mode for the separation of peptides. Within the 310-min analysis, Data Dependent Acquisition Top 20 tandem MS was performed. Raw data analysis and multiple analytical measurements in a single sample were done using Analyst^®^ TF v.1.6 (AB Sciex, USA) software. Precursor and product ion evaluations were completed with PeakView (1.2, AB Sciex). Generated peak lists containing MS and MS/MS spectra were used to identify proteins, protein isoforms, and their modifications with ProteinPilot 4.5 Beta (AB Sciex, USA). Identification was made using the UniProtKB-based reference library of resistant strain *A. baumannii* BAA 1710 (UP000002446) and non-resistant strain *A. baumannii* 17978 (UP000006737). During identifying proteins, the false discovery rate was determined to be 1%, and proteins containing at least two identified peptide fragments were considered correct identification.

### Bioinformatic evaluations

Identified peptides were grouped with Venn diagrams. Linear B Cell Epitope Prediction tool of Immune Epitope Database (IEDB) was used to predict the epitope from identified amino acid sequences (23). The Bepipred Linear Epitope Prediction method was used, and the window size was determined as seven amino acids during calculations (24). Beta-barrel structure of proteins was analyzed by PHYRE2 (Protein Homology/analogY Recognition Engine 2.0) web-based software (25). The BOCTOPUS database was used to determine both beta-barrel structure and transmembrane properties of proteins (26). As another transmembrane site–determining web-based tool, TMHMM 2.0 (Transmembrane Hidden Markov Model) was also used to analyze the transmembrane sites of proteins (27). To analyze the solubility of proteins, PROSO II software was used (28). The potential glycosylation of nine selected proteins was predicted using the GlycoPP v1.0 web server (29).

FASTA sequences of eight intact proteins and one with the missing region (B0V885) were submitted to Alphafold v2.1.0 (30) to generate the three-dimensional (3D) structural models of the respective proteins with default parameters. We selected three models for each run to get the best ranking models. Python script provided by Mayachemtools (31) was used to produce Ramachandran plots of the predicted structures, and we performed all the computations on our local High Performance Computer (HPC) cluster.

## Results

**Figure 1:**
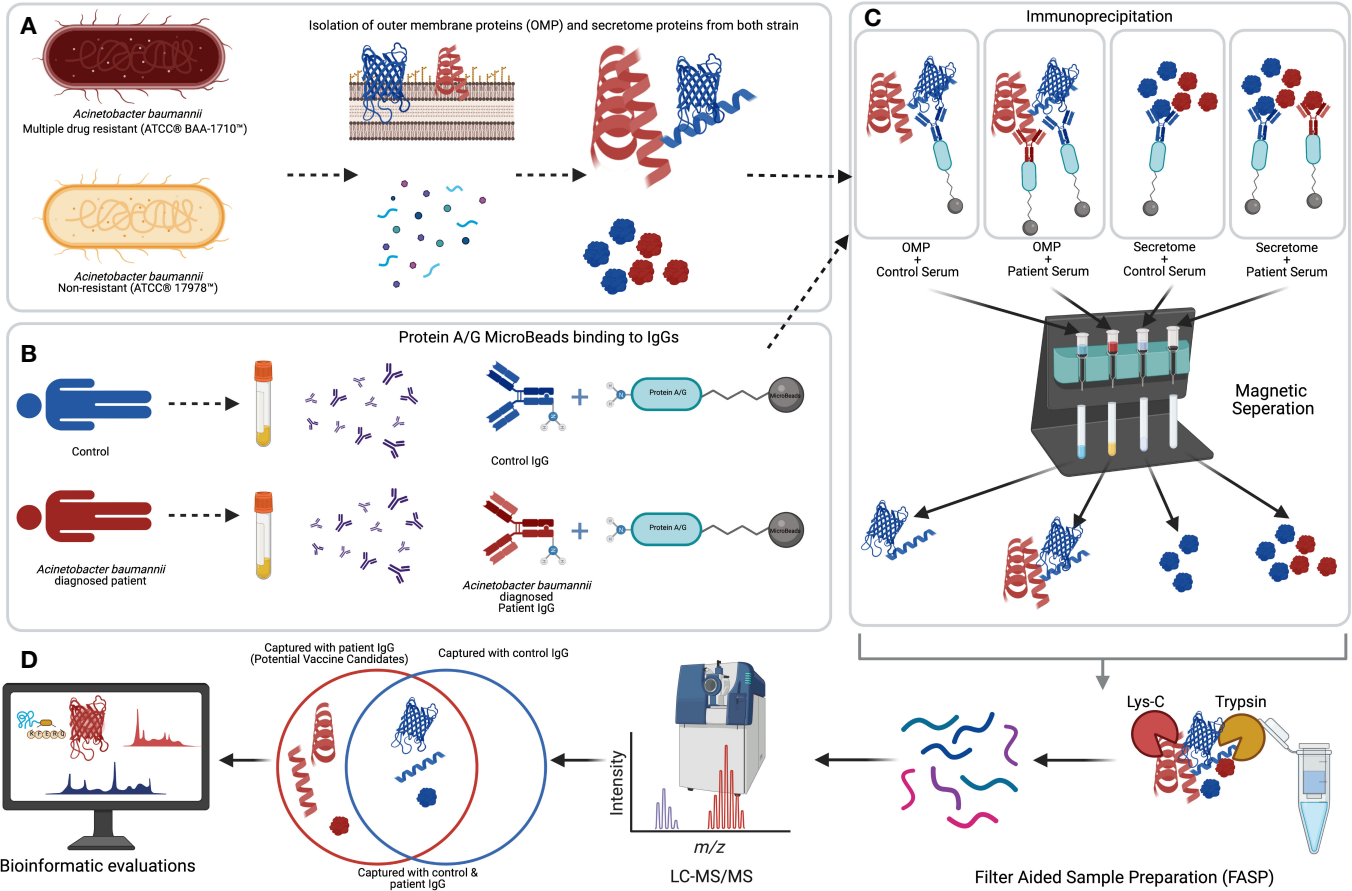
The workflow of our experimental strategy. **(A)** Isolation of the outer-membrane and secretome proteins of *Acinetobacter baumannii*–resistant (BAA-1710) and –sensitive (17978) strains. **(B)** IgGs from human sera were linked to protein A/G microbeads. **(C)** Protein A/G–bound IgG was utilized to bait immunodominant *A. baumannii* proteins from each group. **(D)** Sample preparations, LC-MS/MS analysis, and bioinformatic evaluations of the identified antigenic proteins. Created with BioRender.com.

In this study, we have used blood sera of 29 A*. baumannii*–infected ICU patients. As control, we used 13 individuals treated in the same ICU with no *A. baumannii* infection or other bacterial infection. In addition, three healthy individuals were considered the external control group to eliminate the conserved antigens from normal flora members. Bacterial OMPs and secretome proteins were isolated from multiple drug-resistant (BAA-1710) and non-resistant (ATCC 17978) *A. baumannii* strains. These isolated proteins were subjected to immunoprecipitation with human sera. To capture mutual/conserved antigens, we used the standard ATCC bacterial strains instead of unmapped local clinical isolates. To minimize biological variation between individuals, the sera of the same blood type were combined before immunoprecipitation. The workflow of our experimental strategy is depicted in **Figure 1**.

### Identified subcellular immunogenic proteins of *A. baumannii*

**Figure 2:**
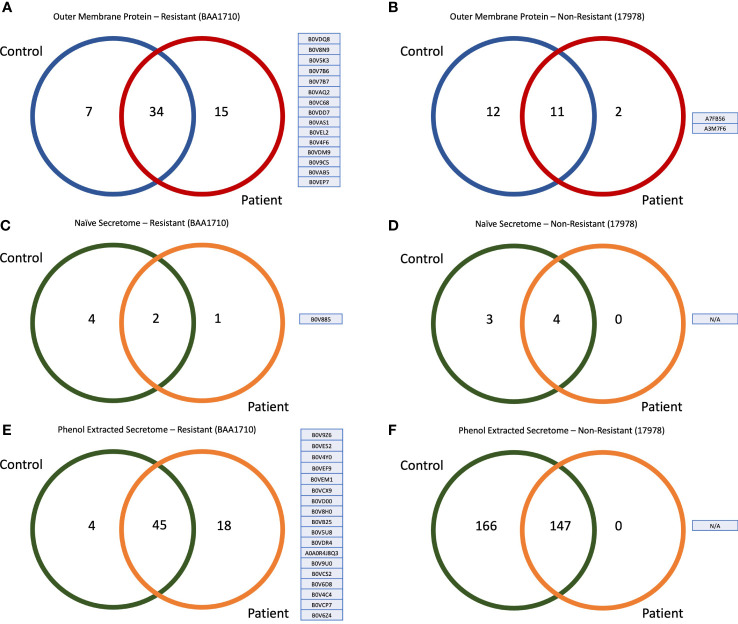
Venn diagram and accession numbers of the identified proteins from diverse groups. **(A)** Fifteen outer-membrane proteins of resistant strain captured *via* patient sera and their accession numbers. **(B)** Two outer-membrane proteins of sensitive strain captured *via* patient sera and their accession numbers. **(C)** One secretome protein of resistant strain that captured *via* patient sera without any enrichment protocol and its accession number. **(D)** No antigenic secretome protein of sensitive strain was captured with patient sera in naïve form. **(E)** Eighteen secretome proteins of resistant strain captured *via* patient sera after enrichment with phenol extraction and their accession numbers. **(F)** No antigenic secretome protein of sensitive strain was captured with patient sera after phenol extraction.

LC-MS/MS analysis, followed by immunoprecipitation with patient sera, revealed 49 OMPs of resistant strain (BAA1710). When immunoprecipitation was performed with control sera, 41 immunogenic proteins were identified for the same strain. Thirty-four of these were commonly identified from control and patient sera, whereas 15 antigens were specifically captured only with patient sera and accession numbers presented in **Figure 2A**. To reason these 15 proteins specifically to the resistant strain, we have mapped the sensitive strain (17978); we identified 23 OMPs by immunoprecipitation with control sera and 13 proteins with patient sera. A comparison of these two groups showed that only two of these proteins were specifically precipitated with patient sera **(** **Figure 2B** **)**, but none of these proteins were shared with non-resistant strains finding; therefore, 15 immunogenic OMPs were classified only to the resistant strain.

We immunoprecipitated bacterial proteins directly from the conditional medium, without any protein enrichment protocols (i.e., phenol and Trichloroacetic acid (TCA)-acetone precipitation), from both control and patient sera. Accomplished LC-MS/MS analysis revealed that only one immunogenic naïve secretome protein of the resistant strain was captured and identified with patient sera (**Figure 2C**). At the same time, there was no identified protein from the naïve secretome of the sensitive strain (**Figure 2D**). The only protein captured was B0V885. This made us think that the protein in conditioned media and/or the IgGs raised against this protein should be abundant. Therefore, we were capable of capturing it without any pre-processes. Because very few proteins were identified from naïve secretome, we concentrated secretome proteins with the phenol-chloroform precipitation. By immunoprecipitation phenol-precipitated secretome proteins with patient sera, we identified 18 proteins specific to the resistant strain (**Figure 2E**), but there were no proteins of the sensitive strain (**Figure 2F**).

Thirty-four antigenic proteins were identified from the outer membrane and secretome (naïve and phenol precipitated) *via* immunoprecipitation with patient sera, explicitly belonging to the resistant strain **(** **Table 1** **)**. The majority of the identified proteins were bacterial membranes and periplasm. When evaluating their molecular function, some molecules function as membrane transporter, symporter, ion binding, antibiotic binding, outer-membrane assembly, and protein secretion. In addition, some molecules have enzymatic activity, like hydrolase, oxidoreductase, and cis-trans isomerase.

**Table 1:** List of the 34 identified resistant *A. baumannii* outer-membrane and secretome proteins, their molecular mass (Da), amino acid lengths, encoding genes, cell localization, and annotated functions.

No	Accesion number	Protein name	Mass (DA)	Length	Gene	Localization	Function
1.	B0VDQ8	Alpha-ketoglutarate permease	49,270	443	*kgtP*	Membrane	Transmembrane transporter activity
2.	B0V8N9	Peptidoglycan D,D-transpeptidase FtsI	67,659	610	*Fts1*	Cell inner membrane	Penicillin binding
3.	B0V5K3	Copper resistance protein B	33,583	300	*copB*	Cell outer membrane	Copper ion binding
4.	B0V7B6	Autotransporter assembly factor TamA	101,763	906	*ABAYE1429*	Cell outer membrane	–
5.	B0V7B7	**Uncharacterized protein**	164,226	1,501	*ABAYE1430*	Membrane	Protein secretion
6.	B0VAQ2	DUF4142 domain-containing protein	22,071	197	*ABAYE2524*	–	–
7.	B0VC68	Glutamate/aspartate transport protein (ABC superfamily, peri_bind)	32,030	297	*gltI*	–	–
8.	B0VDD7	Putative ferrous iron transport protein B (FeoB)	67,515	617	*ABAYE3526*	Integral component of membrane	Ferrous iron transmembrane transporter activity
9.	B0VAS1	**Uncharacterized protein**	11,247	105	*ABAYE2520*	Integral component of membrane	–
10.	B0VEL2	PHB domain-containing protein	31,060	284	*ABAYE0724*	Integral component of membrane	–
11.	B0V4F6	Outer membrane protein	51,536	469	*adeC*	Cell outer membrane	Efflux transmembrane transporter activity
12.	B0VDM9	Tim44 domain-containing protein	34,253	325	*ABAYE0855*	Integral component of membrane	–
13.	B0V9C5	Putative sodium:solute symporter	61,830	578	*ABAYE0188*	Membrane	Symporter activity
14.	B0VAB5	Putative bifunctional protein	115,311	1,071	*ABAYE2663*	Membrane	Hydrolase activity, acting on glycosyl bonds
15.	B0VEP7	Putative amino-acid transport protein	53,450	491	*ABAYE0689*	Cell inner membrane	Alanine:sodium symporter activity
16.	B0V885	Putative outermembrane protein exposed to the bacterial surface	93,798	974	*ABAYE3068*	–	–
17.	B0V9Z6	Putative PQQ-dependent aldose sugar dehydrogenase	–	–	*ABAYE0052*	–	Oxidoreductase activity
18.	B0VE52	**Uncharacterized protein**	44,644	379	*ABAYE3478*	–	–
19.	B0V4Y0	Thiol:disulfide interchange protein	26,405	236	*ABAYE0657*	Periplasm	Required for disulfide bond formation in some periplasmic proteins.
20.	B0VEF9	**Uncharacterized protein**	812,466	8,200	*ABAYE0792*	–	–
21.	B0VEM1	Putative long-chain fatty acid transport protein	50,578	476	*ABAYE0711*	–	–
22.	B0VCX9	YceI domain-containing protein	21,042	196	*ABAYE2114*	–	–
23.	B0VD00	Chaperone SurA	49,578	441	*surA*	Periplasm	Peptide binding
24.	B0V8H0	Outer membrane protein assembly factor BamD	42,829	385	*bamD*	Cell outer membrane	Gram-negative-bacterium-type cell outer membrane assembly
25.	B0VB25	**Uncharacterized protein**	15,778	144	*ABAYE2389*	–	–
26.	B0V5U8	Putative membrane-bound lytic murein transglycosylase	46,973	430	*ABAYE3869*	–	–
27.	B0VDR4	**Uncharacterized protein**	366,110	3,369	*ABAYE0821*	–	Calcium ion binding
28.	A0A0R4J8Q3	Peptidyl-prolyl cis-trans isomerase	25,601	235	*fklB*	Membrane	Peptidyl-prolyl cis-trans isomerase activity
29.	B0V9U0	**Uncharacterized protein**	27,620	241	*ABAYE0130*	Cell outer membrane	–
30.	B0VCS2	**Uncharacterized protein**	46,896	408	*ABAYE0968*	–	–
31.	B0V6D8	TolA_bind_tri domain-containing protein	32,499	294	*ABAYE1588*	–	Protein trimerization
32.	B0V4C4	**Uncharacterized protein**	36,121	325	*ABAYE1860*	–	–
33.	B0VCP7	Putative protease	104,035	920	*ABAYE0990*	–	Metal ion binding
34.	B0V6Z4	Putative toluene tolerance protein (Ttg2D)	24,332	219	*ABAYE0388*	–	–

### B-cell epitope prediction

We used a sequence-based epitope prediction tool, IEDB, to predict identified protein B-cell epitopes. Outputs of this tool showed us that B0V885 and B0VAB5 have highly scored epitope regions. Moreover, the rest of the immunogenic proteins had a non-negligible epitopic region score (**Supplementary Figure 1**).

### Structural and solubility predictions of the identified proteins

Predictions by BOCTOPUS revealed that nine of the identified proteins have transmembrane beta-barrel structure topology. These molecules were B0V4F6, B0V5K3, B0V7B6, B0V7B7, B0V9C5, B0V9U0, B0VCP7, B0VE52, and B0VEM1. These phenomena were evaluated with PHYRE2 and showed us that B0V7B6, B0VCX9, B0VEM1, and B0V9U0 have beta-barrel structures. By viewing two different logics, we could identify 13 of the 33 proteins demonstrating beta-barrel structure, presumably insoluble. One of the identified proteins, B0VEF9, had a quite long amino acid length (8,200 aa); we could not get any prediction within the limitation of both tools. We also evaluated the transmembrane properties of the identified proteins. TMHMM tool provided us that B0VDQ8, B0V8N9, B0VDD7, B0VDM9, B0V9C5, and B0VEP7 had ≥2 transmembrane regions. In addition, the solubility scores of the identified proteins assessed with PROSO II and those with a score above 0.6 were accepted as soluble (**Supplementary Table 2**). Of the 34 proteins, only nine were above the solubility threshold with the default parameters (B0V7B7, B0VC68, B0V4F6, B0VAB5, B0V9Z6, B0VE52, B0VD00, B0V8H0, and A0A0R4J8Q3).

### Possible candidates for recombinant vaccine production

**Figure 3:**
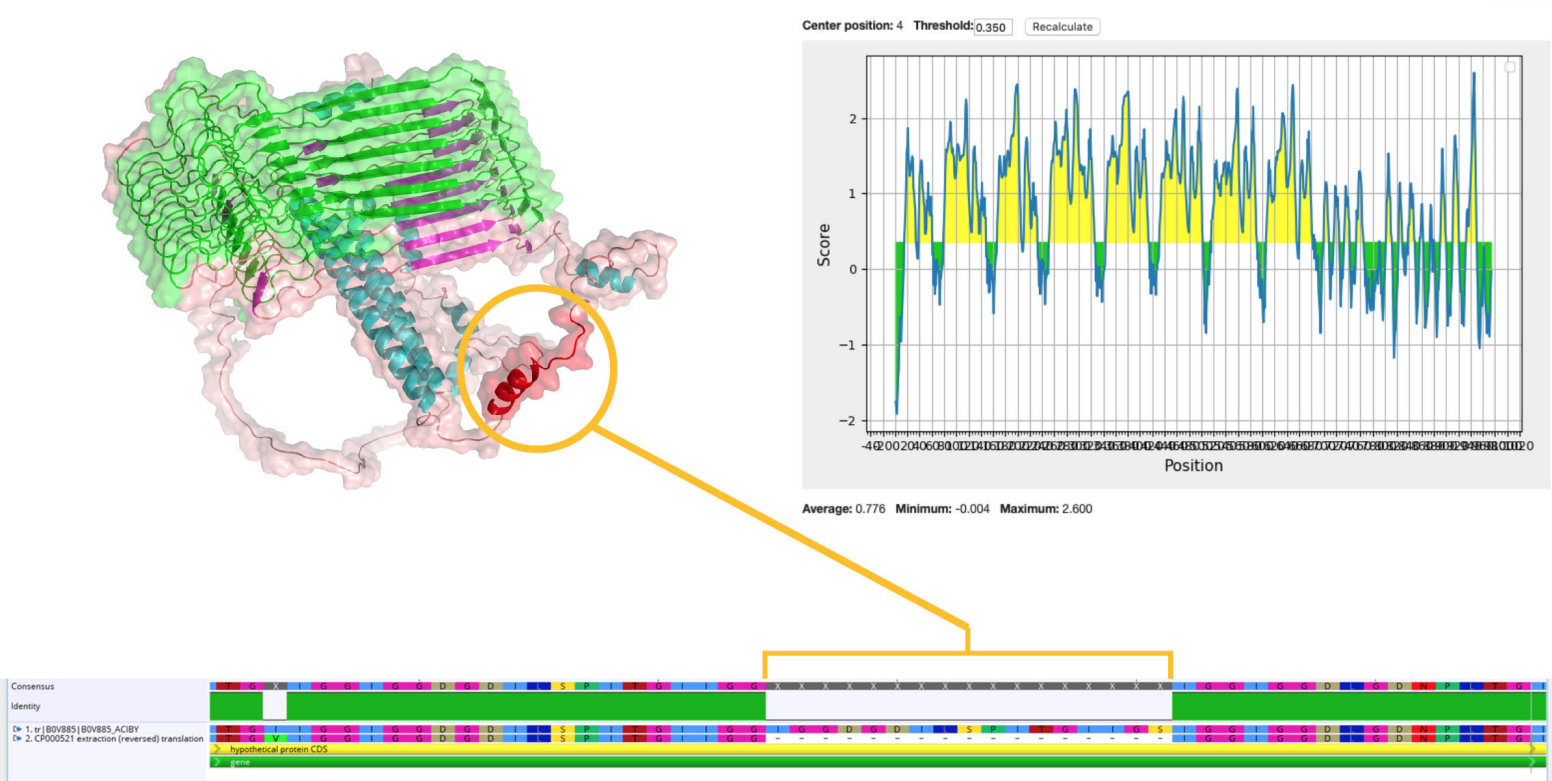
Predicted epitope regions and 3D structure of B0V885 protein. Circled red helix was a 17–amino acid length region in the resistant strain, whereas it was not present in the sensitive strain.

On the basis of the structural analysis and solubility scores, we mainly focused on eight predicted soluble proteins for a candidate. In addition, we evaluated the rest of our list of proteins on immunogenicity and epitope predictions. Although B0V885 was detected as insoluble by PROSO II, when we scrutinized IEDB outputs, it was the highest epitope conveying protein. B0V885 was uniquely captured from naïve secretome, without any enrichment, in the native conformational state. Furthermore, when the reference genomes of resistant and sensitive strains were aligned, a noticeable peptide region (between the 735th and 752nd amino acids of B0V885 protein) was present in the resistant strain but not in the insensitive strain (**Figure 3**). Above all, B0V885 has attracted our attention as a druggable protein. Therefore, our recombinant vaccine candidate list rolled up to nine. Epitope analysis, beta-barrel, and transmembrane topology of the identified proteins were depicted in **Supplementary Figures 1**-**3**. Because O-linked glycosylation could boost the antigenicity of proteins, we would like to overview possible glycosylation sites of the candidate proteins. According to GlycoPP v1.0, all nine selected proteins had several potential glycosylation sites (**Supplementary Table 3**).

### Epitope alignment to protein structure

**Figure 4:**
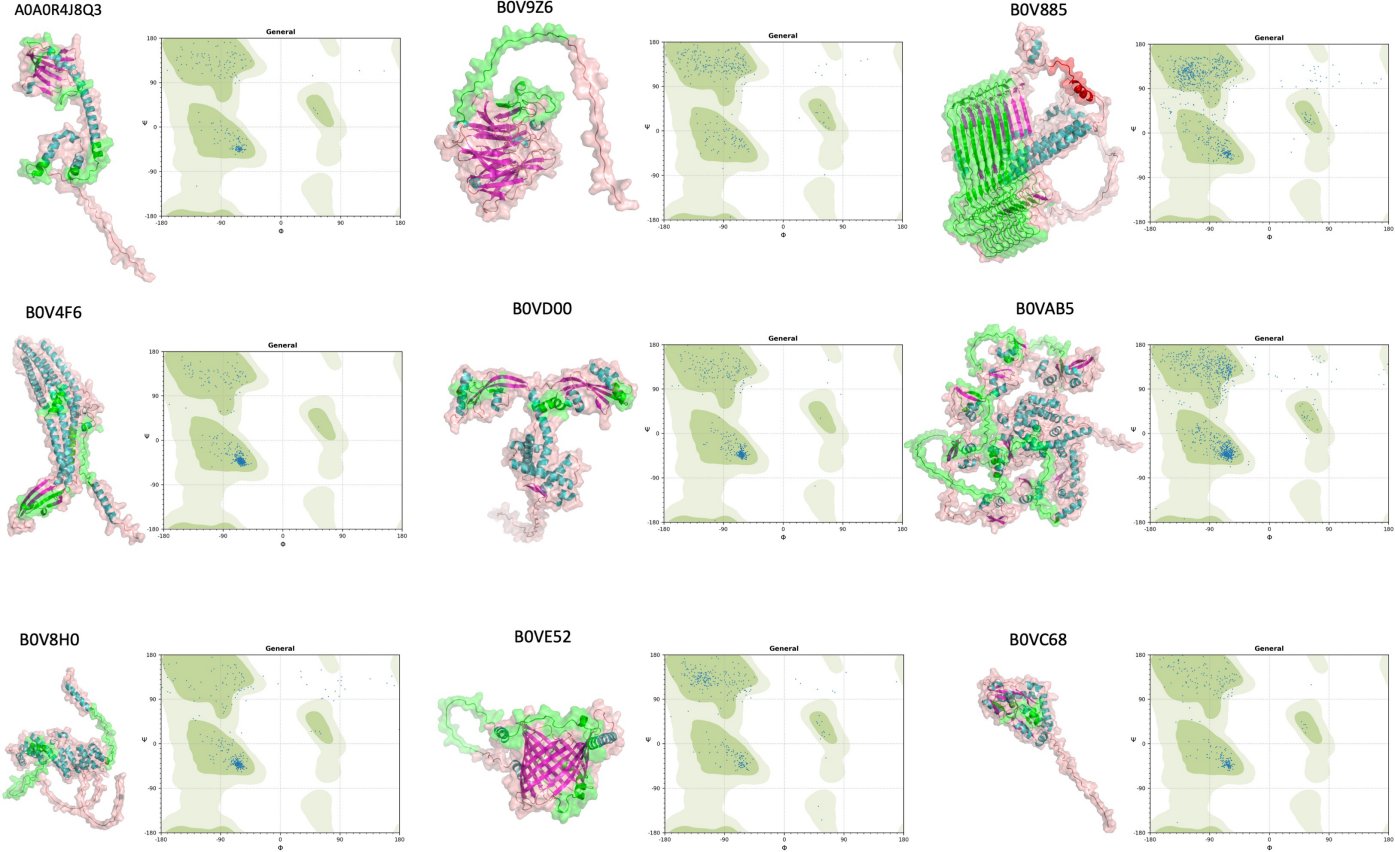
Tertiary structure prediction of nine selected proteins (A0A0R4J8QA3, B0V9Z6, B0V885, B0V4F6, B0VD00, B0VAB5, B0V8H0, B0VE52, and B0VC68) by AlphaFold v2.1.0. IEDB predicted B-cell epitope regions were overlapped and highlighted with green labeling. Ramachandran plots of these proteins were also depicted for the presentation of alpha-helix and beta-sheet distribution.

The expectation was that an epitope was valid if it was located on an accessible region of a protein. Therefore, we wondered whether the selected nine proteins fit into this criterion. In state-of-the-art visualization of the candidate proteins, we employed AlphaFold2. We successfully calculated 3D models of our nine proteins (**Figure 4**). On the 3D models of our proteins, selected epitopic regions (e.g., length of the epitopic region and possible O-linked glycosylation) were highlighted. In addition, Ramachandran plots were extrapolated to verify AlphaFold2 generated 3D models if it was in the favorable areas of the plots. AlphaFold2 enabled us to model such large proteins, which was beyond the limits of other prediction tools.

## Discussion

Nosocomial infectious diseases cause the death of many people in ICUs worldwide. Among the bacteria that cause intensive ICU, *A. baumannii* has a devastating impact due to multiple antibiotic resistance (32). With the arsenal of antibiotics that target *A. baumannii* failing over time, there is an urgent need to develop vaccines against this species to protect critically ill ICU patients (33–35).

Recently, two main strategies were followed to identify vaccine candidates against *A. baumannii*. One of them was immunoproteomic approaches that use blood sera of *A. baumannii–*infected patients or animal models (36). The other was a computational method based on *in silico* analysis and prediction of the antigenic structure using bacterial genome sequences (25, 37). In the present study, our strategy was to capture and identify the immunodominant antigenic molecules of *A. baumannii via* IgGs from human subjects’ sera. Our experimental strategy relied on the hypothesis that immunoglobulins of infected persons can interact with immunologically dominant bacterial proteins. Subtractive analysis of captured proteins with patient and control immunoglobulins revealed immunologically remarkable molecules, which trigger a *de facto* response of the human immune system against the antibiotic-resistant *A. baumannii*.

Identification of immunogenic proteins using serum immunoglobulins was applied successfully in the past. Recently, gel-based methods followed by MALDI-TOF analysis have been used to identify vaccine candidate proteins (36, 37). A study by Fajardo Bonin et al. (36) employed immunoproteomic approach and identified six membranous proteins: OmpA, Omp34kDa, OprC, OprB-like, OXA-23, and ferric siderophore receptor protein. In the gel-based methods, denaturation of proteins, as opposed to *in vivo*, brings a drawback in antibody-antigen interactions due to loss of conformational epitopes. These approaches were low yielding to determine bacterial antigens in real case scenarios; therefore, we performed immunoprecipitation, which allows us to pull down the proteins in non-denaturing conditions as much as possible.

Other published studies have identified several different proteins, and a great majority of those have focused on the outer membrane and outer-membrane vesicles. OmpA, which has an essential function in biofilm formation and pathogenicity of *A. baumannii*, was conducted in a murine model by Luo et al., (35). In this study, diabetic mice were vaccinated with recombinant OmpA (rOmpA), and 2 weeks later, mice were infected *via* the clinical *A. baumannii* isolate. Vaccination was found to prolong survival of mice and significantly reduce the bacterial load in tissues, as vaccination caused high titers of anti-OmpA antibodies associated with survival in mice model (35). Ata and Bap are the other proteins applied in immunization against *A. baumannii*, yet the prevalence and solubility of produced recombinant protein were the significant challenges for vaccination (34, 38). OmpK and Ompp1 were described as potential vaccine candidates by a reverse vaccinology study published. Chiang et al. (39) found that these antigens were highly immunogenic due to the high production of IgG antibodies after vaccinating (two times) mice with 3 µg of recombinant antigens. Then, they confirmed 60% protection in the murine pneumonia challenge model with the *A. baummannii* and porcine mucin. Another study on clinically isolated strains identified several proteins like CarO-like porin, AdeK, TonB, OmpH, and BamABDE that could be regarded as diagnostic markers (40) and potential protective agents. In a reverse vaccinology study, a dataset of *A. baumannii* whole genomes investigated to identify the new putative vaccine candidates and selected DcaP-like protein and HP-2 as candidates among 11 (41). Overall, there are many different approaches to investigate the vaccine candidates both immunoinformatic and other omics strategies, which are extensively reviewed (42).

Evolutionarily, humans accommodate many bacterial species; some of which are opportunistic pathogens. Classical vaccine candidate identification methodologies usually mislead or are prone to capture conserved proteins, i.e., OMPs. In our methodology, we focused on genuine targets by subtracting those proteins recognized by healthy individuals’ IgGs. Using a subtractive approach, we identified 34 unique proteins *via A. baumannii*–infected patient sera in the membranous and secretome of these bacteria. Most of those proteins were not deeply analyzed with classical or *in silico* approaches and were somehow disregarded as potential vaccine candidates (e.g., B0V885/ABAYE3068) (25, 43, 44). Considering the structure, transmembrane properties, epitope regions, and solubility of the identified proteins, we further analyzed nine of them as potential recombinant vaccine candidates.

One of these proteins was B0VC68 (glutamate/aspartate transport protein), a member of the ABC superfamily, previously described as a periplasmic protein with an increased expression in tetracycline response conditions (45) and acid tolerance (46). Identification of B0VC68, specifically in antibiotic-resistant strains, and its relation with tetracycline response make this protein a valuable target for vaccine development and small-molecule targeting studies. B0V4F6 (AdeC) is another OMP that functions as efflux transmembrane transporter activity. It has been reported that AdeC has a vital role in drug efflux and extrusion of compounds (47) and was known as necessary in the maintenance of antibiotic resistance. Ni et al. (48) suggested AdeC as a vaccine candidate in a reverse vaccinology study. Contradictory to its function as an efflux pump and structural predictions, our initial analysis with standard bioinformatical tools (BOCTOPUS, TMHMM, and PROSO II) found AdeC to be a soluble protein without any transmembrane region. Further structural prediction by AlphaFold2 confirmed that this AdeC is a transmembrane protein; therefore, it cannot be considered as a recombinant vaccine candidate. B0VE52 has also been listed as soluble proteins by the standard prediction tools, and it has been suggested as a candidate for the *A. baumannii* vaccine *in silico* approaches (25). Our structural prediction *via* AlphaFold2 led us that the B0VE52 was another unfortunate candidate with a beta-barrel structure. Relying on the classical linear sequence-based prediction approaches that have the potential to mislead outcomes and state-of-the-art 3D structural prediction tools should be taken into consideration.

Another protein identified from the secretome, BamD (B0V8H0), is a part of the membrane protein assembly complex that inserts beta-barrel proteins into the outer membrane. Although no study on the immune potential of BamD was found in the literature, it has been reported that immunization with BamA, a partner of BamD in establishing membrane assembly, increased survival to 60% in the murine model (49). A0A0R4J8Q3 (FklB), which functions as peptidyl-prolyl cis-trans isomerase, was another identified protein with our approach. According to gene ontology analysis, this membrane annotated protein has a role in the protein folding process. FklB was reported previously as a vaccine candidate by Chiang et al. (39) and proved that the protein is highly immunogenic. Like FklB, SurA (B0VD00), which has a role in OMP folding and assembly, was captured and identified in our study. Functional similarities with FklB (50) and interactions with BamA (49) may indicate that this protein is a potential vaccine candidate. B0V9Z6 was a putative PQQ-dependent aldose sugar dehydrogenase. Its oxidoreductase function was identified from the secretion of A. *baumannii* and had a significant B-cell epitope region. According to our structural model, predicted epitopes are located on the accessible part of the protein, making it more likely a protein vaccine candidate. To our knowledge, there was no record of this protein’s immunogenicity and protective role in any model.

Immunoprecipitation identified protein from the membranous structure was B0VAB5 (putative bifunctional protein), a large protein consisting of 1,071 amino acids with lytic transglycosylase and hydrolase activity. According to gene ontology annotations, this protein has a role in the peptidoglycan metabolic process. Having LysM repeat regions implies that this protein is a cell wall–binding protein and is located on the accessible surface of the bacteria (51). Epitope predictions provide us with a considerable number of epitopic regions. Overlapped epitope prediction and 3D structural analysis showed us that predicted epitopes were placed on the accessible parts of protein that is crucial for a vaccine candidate. To our best knowledge, this protein has not been reported as a vaccine candidate for *A. baumannii.* Although there is no evidence in the literature about this entity, our experimental approach allowed us to identify B0VAB5 as an immunogenic protein that is directly captured *via* infected patient IgGs.

Among the identified proteins, B0V885 caught our attention by having several epitopic regions and physically being a part of the secretome. Interestingly, it was the only protein that could be immunoprecipitated directly from conditioned media without further enrichment. This suggests that B0V885 was a highly abundant and immunodominant protein, therefore easy to detect *via* immunoproteomics. B0V885 (putative membrane protein exposed to the bacterial surface) consists of 974 amino acids. Epitope analysis of B0V885 presented that the protein has eight significant, mostly repeated epitope regions between 79th and 656th amino acids. Structural evaluations by AlphaFold2 showed us this protein consisted mainly of β-sheets, and our predicted epitope regions fall within this structurally unique part rather than the relatively small, helical part of the protein. When the whole amino acid sequence was analyzed, the solubility of intact protein was below the solubility threshold according to PROSO II. Nevertheless, a partial analysis of repeated epitopic regions was soluble. Furthermore, the GlycoPP tool suggested that those repeated units have a high tendency to O-glycosylation, which might enhance the antigenicity and solubility of a protein. The presence of an insoluble protein in the secretome might be evidence that this protein could be conducted *via* extracellular vesicles. This phenomenon might also explain why this protein is only captured from naïve conditioned media. Interestingly, the comparison of the reference sequences of B0V885 between antibiotic-sensitive and antibiotic-resistant strains revealed the presence of an additional 17 amino acids residue in the resistant strain. The presence of this peptide sequence might affect the unknown function of this protein, and it might be related to drug resistance. Further studies are needed on this protein structure, function, and its relationship with drug resistance. Because most of conducted *in silico* analysis were relied on linear sequence-based epitope prediction algorithms, they failed to find conformational epitopes and antibody interaction regions. For instance, B0V885 has eight small tandemly repeating epitopes according to amino acid sequence–based prediction tools. When we overlapped sequence-based epitope information and 3D model, topologically, this eight-epitope region stands together within close vicinity as part of a unified interaction region exposed to accessible site of protein. Therefore, there is a need for the advance epitope prediction tools for accurate prediction from 3D structures of a subject protein.

Using immunoproteomics approach, we have identified 34 proteins; among them, we focused on nine proteins based on their solubility and structural analysis. The rest of the unconsidered proteins may still be revisited for their potential to be a vaccine candidate. In a recent study, *A. baumannii* BauA protein was fused to C-lobe of Neisseria meningitidis transferrin binding protein B (TbpB) and elicited between 50% and 100% protection in mice (18). Novel hybrid and chimeric antigen technologies could be applicable for those disregarded proteins due to their low solubility.

In conclusion, our study provided data about remarkable vaccine candidates after comprehensive proteomics and bioinformatic analyses. Capturing and identifying the bacterial proteins with patient immunoglobulins provide *de facto* vaccine candidates because these molecules can trigger an immune response and establish immunity against the pathogen. By the nature of immunoprecipitation, non-specific bindings may mislead the results, and more stringent evaluations were needed. To test the protective capacity of candidate antigens, *in vivo* challenge experiments should be considered. Here, our methodology unearthed different set of presumably immunogenic proteins from reverse vaccinology and *in silico* approaches. Combining immunoproteomics with new bioinformatic tools could potentially enhance vaccine discoveries.

## Data availability statement

The mass spectrometry proteomics data have been deposited to the ProteomeXchange Consortium via the PRIDE (52) partner repository with the dataset identifier PXD037507.

## Ethics statement

This study was reviewed and approved by Erciyes University Hospital, were collected with the approval of the local Human Ethical committees (ERU_ LEC_2013-445). The patients/participants provided their written informed consent to participate in this study.

## Author contributions

Study design: SO, MD, AUK, GD, SA-G, HG and MA. Blood sample collection: MD and AUK. Sample preparation: SO, SA-G and MA. Proteomic analysis: SO, SA-G, MA and HG Bioinformatic evaluations SO, SA-G, MA and HG Manuscript preparation: SO, MD, AUK, GD, SA-G, HG and MA. All authors contributed to the article and approved the submitted version.

## Funding

This work was supported by The Scientific and Technological Research Council of Türkiye (TUBITAK) (Project Number: 114S571).

## Acknowledgments

We thank Soriene Nazik Ozcan, Elif Abagail Ozcan, and Eugene Steele for language editing.

## Conflict of interest

The authors declare that the research was conducted in the absence of any commercial or financial relationships that could be construed as a potential conflict of interest.

## Publisher’s note

All claims expressed in this article are solely those of the authors and do not necessarily represent those of their affiliated organizations, or those of the publisher, the editors and the reviewers. Any product that may be evaluated in this article, or claim that may be made by its manufacturer, is not guaranteed or endorsed by the publisher.
