# Supplementary material for: A subtractive proteomics approach for the identification of immunodominant Acinetobacter baumannii vaccine candidate proteins
Source: Front Immunol. 2022 Nov 10;13:1001633. doi: 10.3389/fimmu.2022.1001633 (PMC9687788; doi:10.3389/fimmu.2022.1001633)
Supplement: Supplementary file 1 [file DataSheet_1.pdf]

Supplementary Files

Supplementary Table 1A. Demographic characteristics of patients with *A. baumannii* bacteremia and control group

	**Patients (n=29)**	**Control (n=13)**	***p***
Age (mean±ss)	51.5±19.5	63.8±14.3	0.049
Male gender (%)	15 (51.7)	9 (69.2)	0.333

Supplementary Table 1B. Intensive Care Units (ICU) where patient and control groups with *A. baumannii* bacteremia were followed

**Units**	**Patients (n=29)** **n (%)**	**Control (n=13)** **n (%)**	***p***
Internal ICU	12 (41.4)	3 (23.1)	0.573
Anestezia ICU	8 (27.6)	4 (30.8)	
General Surgery ICU	7 (24.1)	4 (30.8)	
Brain Surgery ICU	1 (3.4)	2 (15.4)	
General ICU	1 (3.4)	0 (0.0)	

Supplementary Table 1C. Reasons for hospitalization of patients with *A. baumannii* bacteremia and control groups in Intensive Care Units (ICU)

Reason	Patients(n=29) n (%)	Control (n=13) n (%)
Diabetes mellitus	-	2 (15.4)
Renal failure	-	1 (7.7)
Sepsis	3 (10.3)	1 (7.7)
İntraabdominal infection	2 (6.9)	1 (7.7)
Abdominal surgery	3 (10.3)	2 (15.4)
İntracranial bleeding	1 (3.4)	3 (23.1)
Trauma	4 (13.8)	1 (7.7)
Gastrointestinal bleeding	-	1 (7.7)

Pneumothorax	-	1 (7.7)
Pneumonia	6 (20.7)	-
Toxic epidermal necrosis	1 (3.4)	-
Cerebrovascular disease	2 (6.9)	-
Gullian barre syndrome	1 (3.4)	-
Orthopedic emergencies	1 (3.4)	-
Preecampsia	2 (6.9)	-
ARDS	1 (3.4)	-
Alveolar hemorrhage	1 (3.4)	-
Fornier gangrene	1 (3.4)	-

Immune Epitope DataBase (IEDB) Results:

A0A0R4J8Q3:

Center position: 4 **Threshold:** 0.350 Recalculate

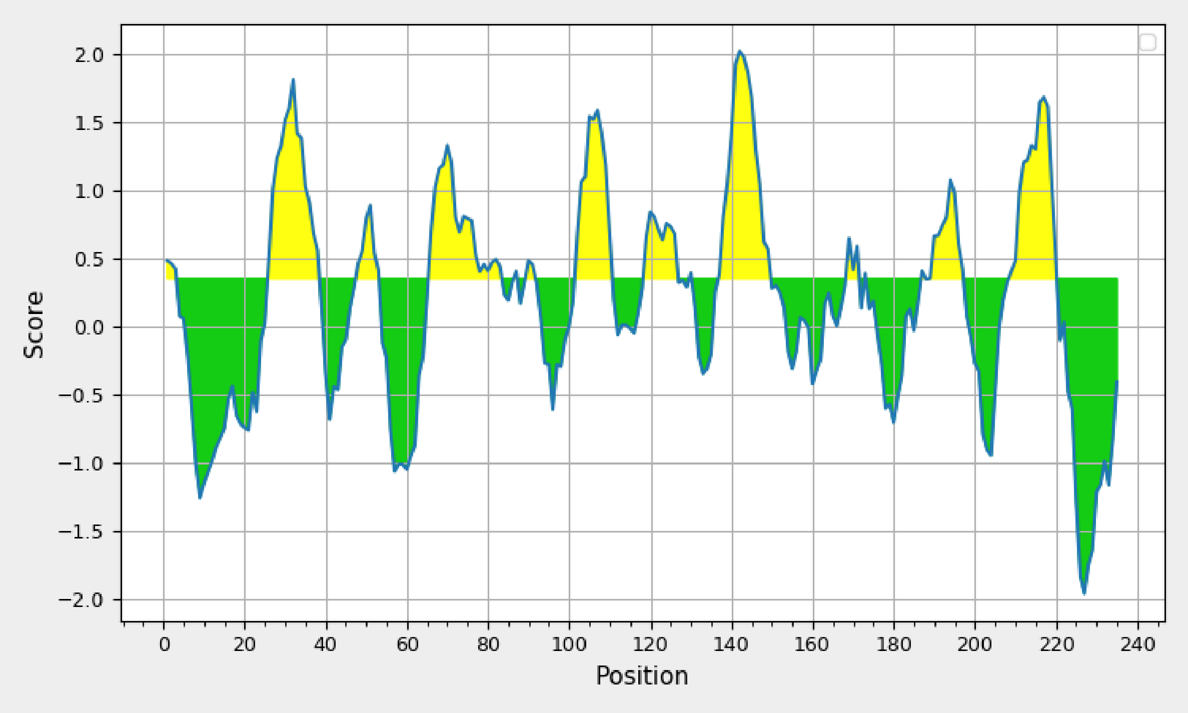

Average: 0.206 **Minimum:** -0.008 **Maximum:** 2.018

B0V4F6:

Center position: 4 **Threshold:** 0.350 Recalculate

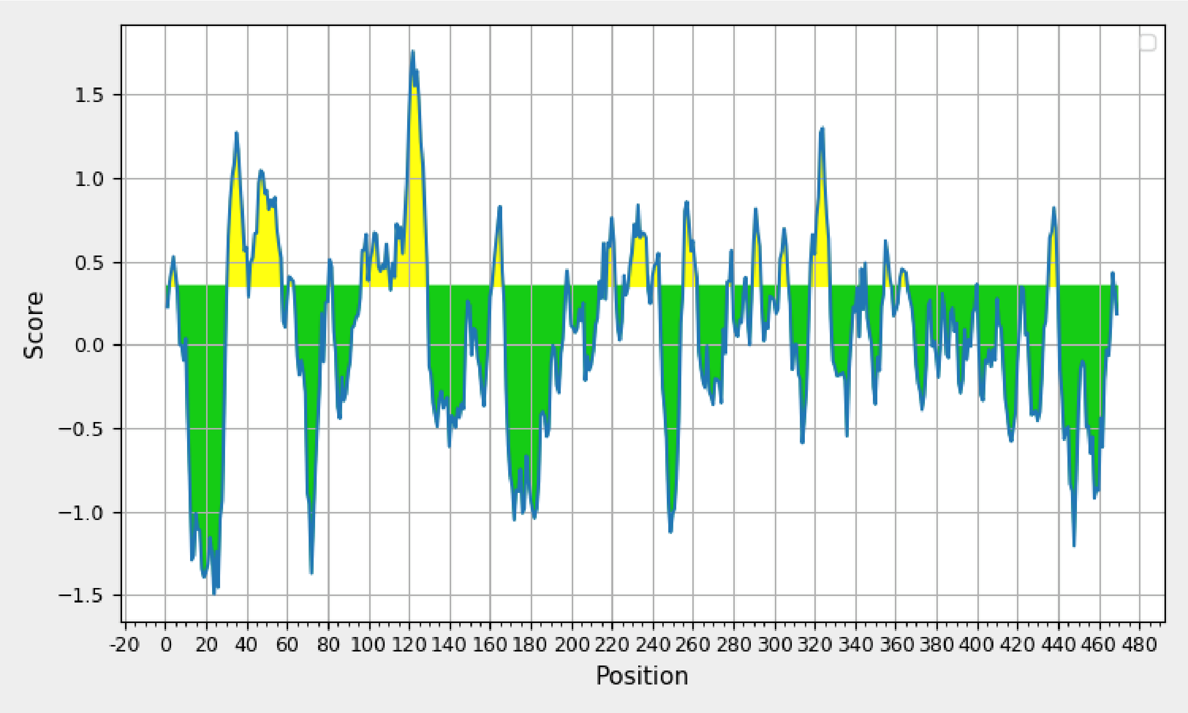

Average: 0.053 **Minimum:** -0.002 **Maximum:** 1.756

B0V8H0:

Center position: 4 **Threshold:** 0.350 Recalculate

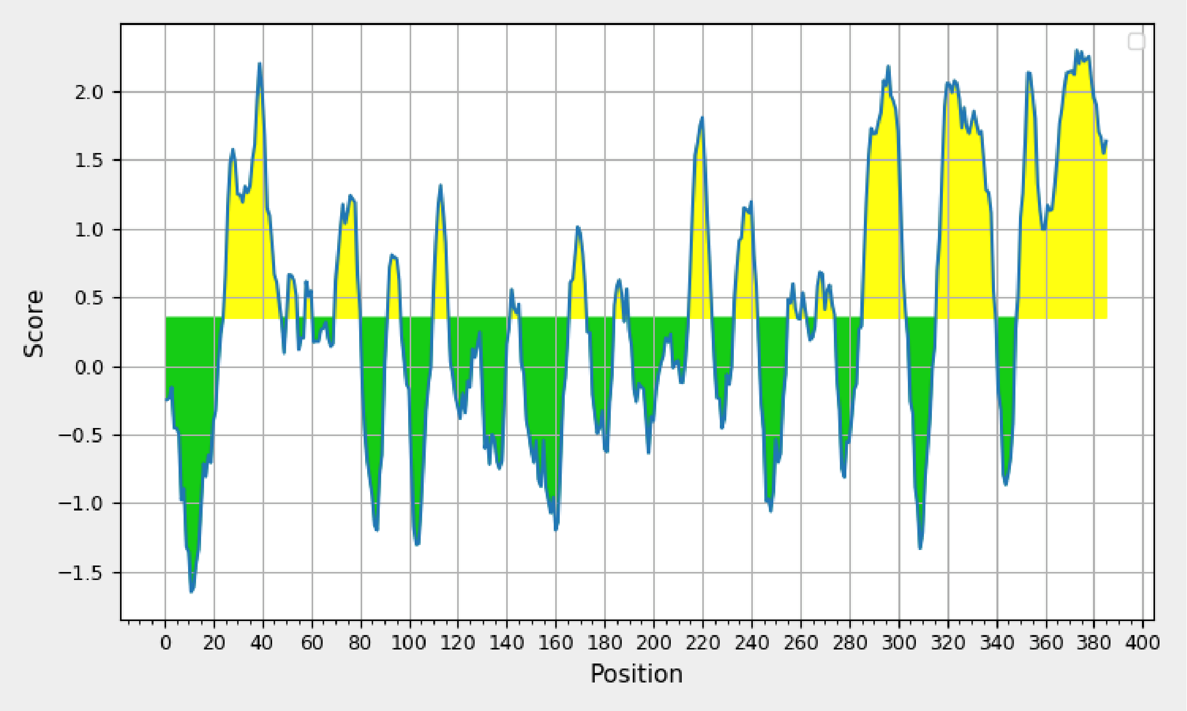

Average: 0.400 **Minimum:** -0.004 **Maximum:** 2.297

B0VC68:

Center position: 4 **Threshold:** 0.350 Recalculate

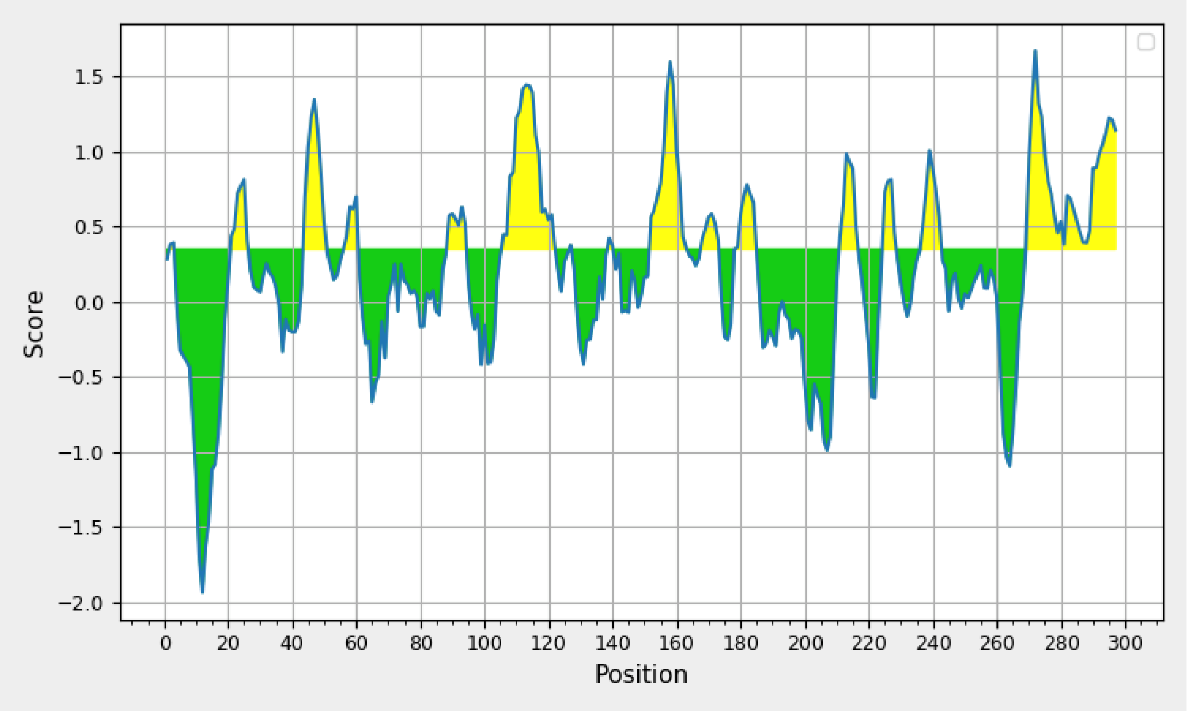

Average: 0.209 **Minimum:** -0.008 **Maximum:** 1.669

B0VE52:

Center position: 4 **Threshold:** 0.350 Recalculate

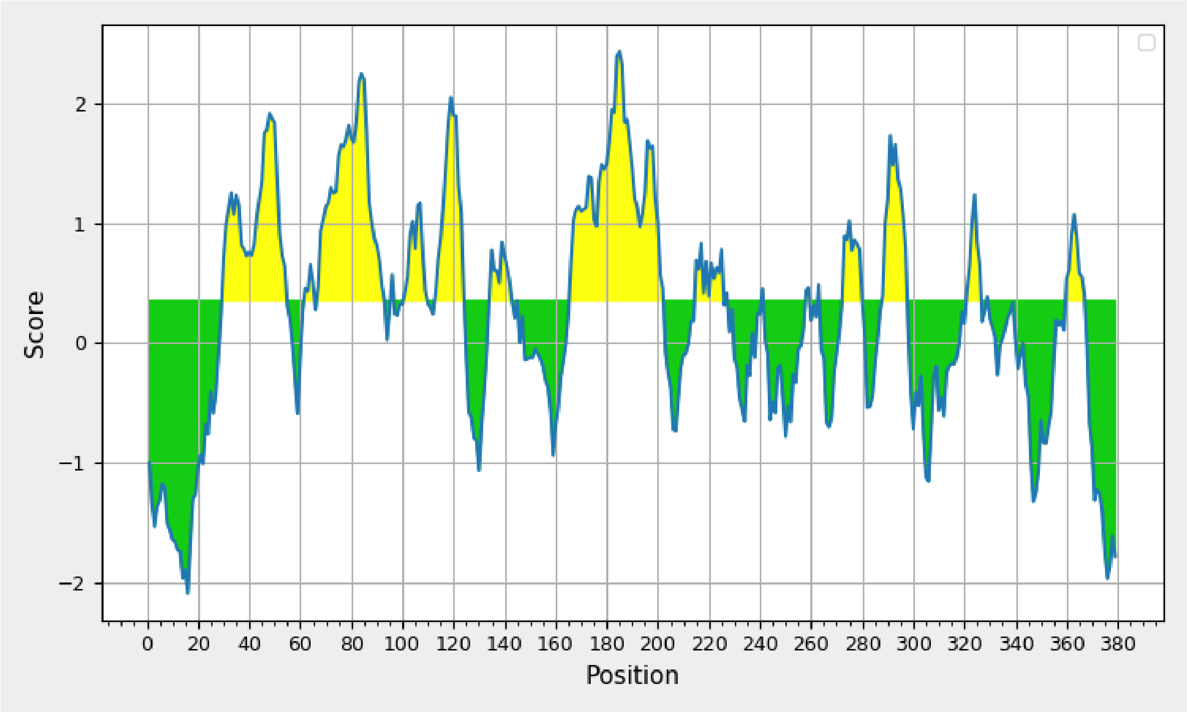

Average: 0.262 **Minimum:** -0.003 **Maximum:** 2.432

B0V9Z6:

Center position: 4 **Threshold:** 0.350 Recalculate

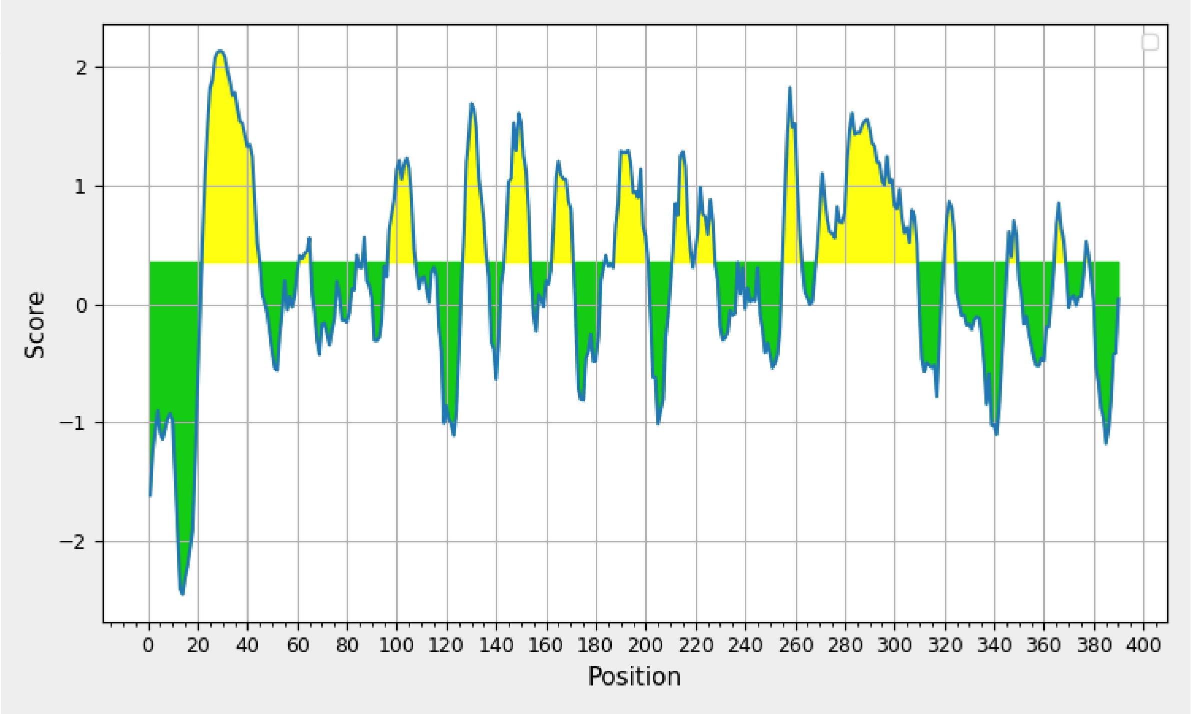

Average: 0.255 **Minimum:** -0.003 **Maximum:** 2.136

B0VD00:

Center position: 4 **Threshold:** 0.350 Recalculate

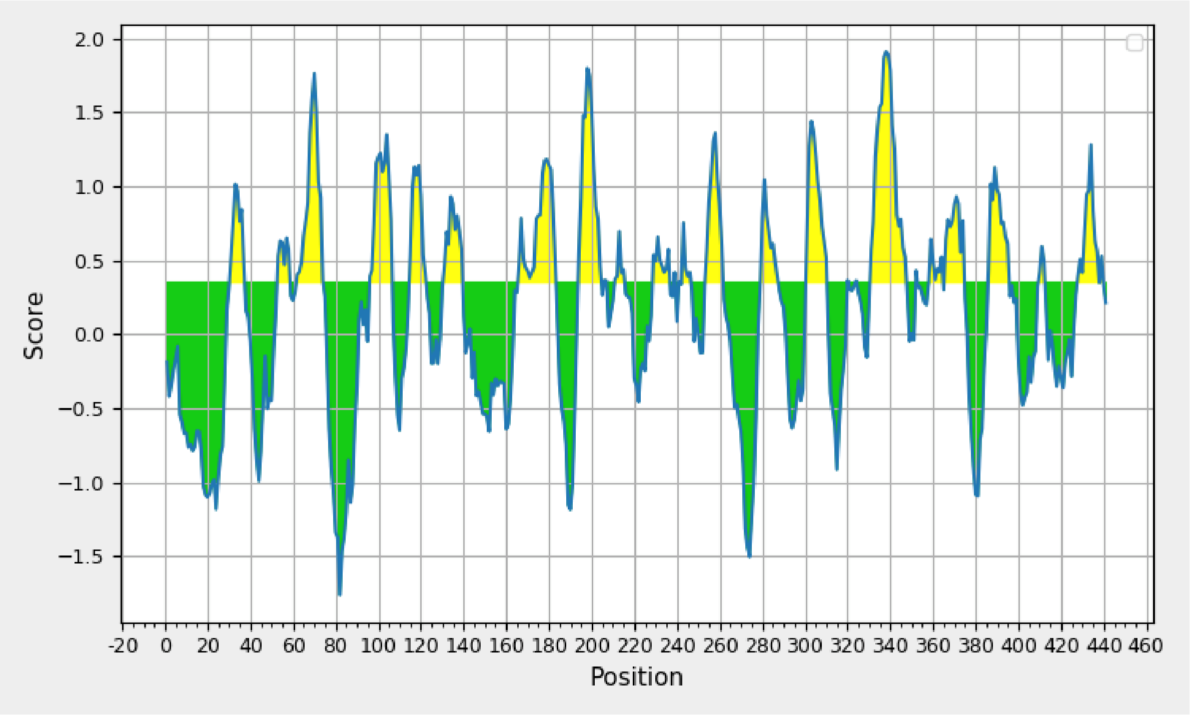

Average: 0.181 **Minimum:** -0.021 **Maximum:** 1.908

B0V885:

Center position: 4 **Threshold:** 0.350 Recalculate

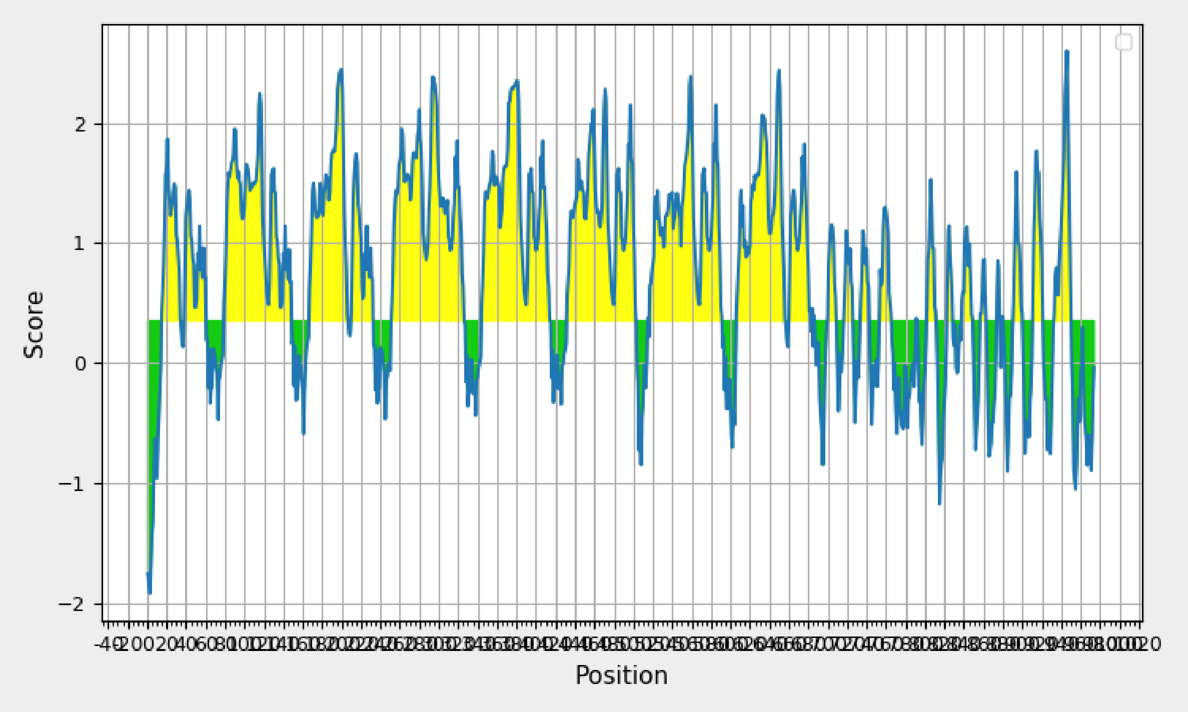

Average: 0.776 **Minimum:** -0.004 **Maximum:** 2.600

B0VAB5:

Center position: 4 **Threshold:** 0.350 Recalculate

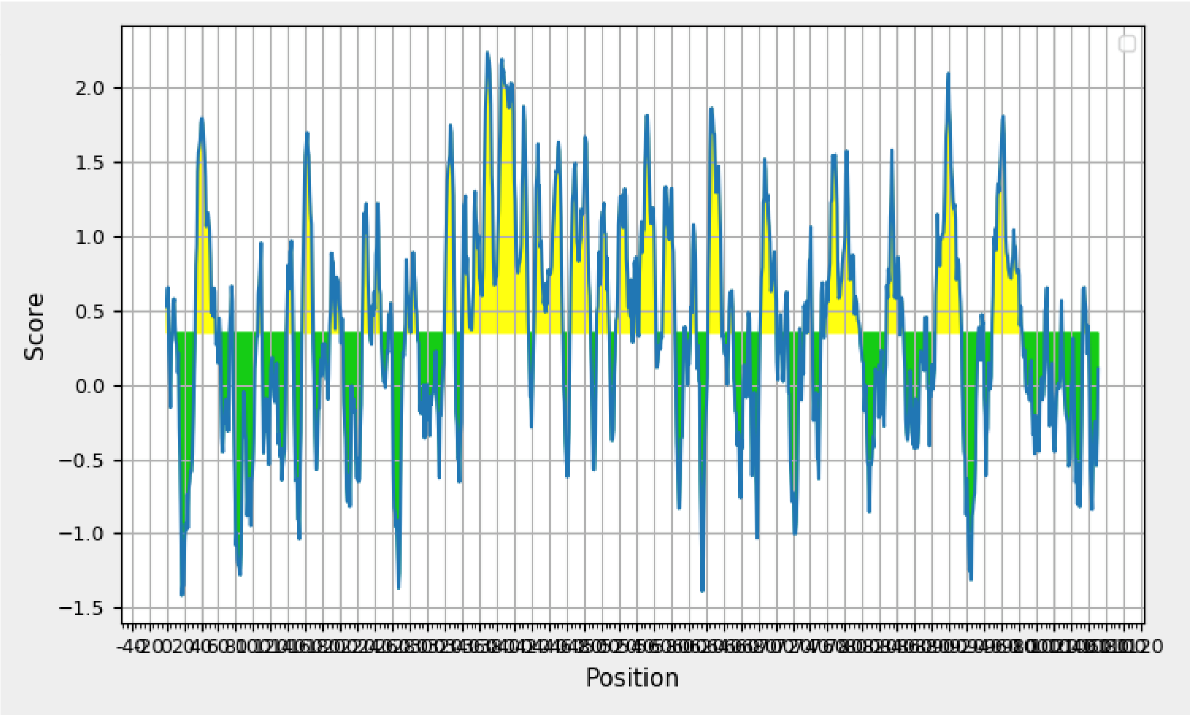

Average: 0.384 **Minimum:** -0.001 **Maximum:** 2.237

Supplementary Figure 1. Immune Epitope Database (IEDB) results of nine selected proteins. Analysis was done by using default parameters of the B-Cell epitope prediction tool Bepipred Linear Epitope Prediction 2.0. The threshold value was 0.35; window size was chosen as seven means center position four and the epitope regions of selected proteins were depicted under accession numbers.

BOCTOPUS Results

A0A0R4J8Q3:

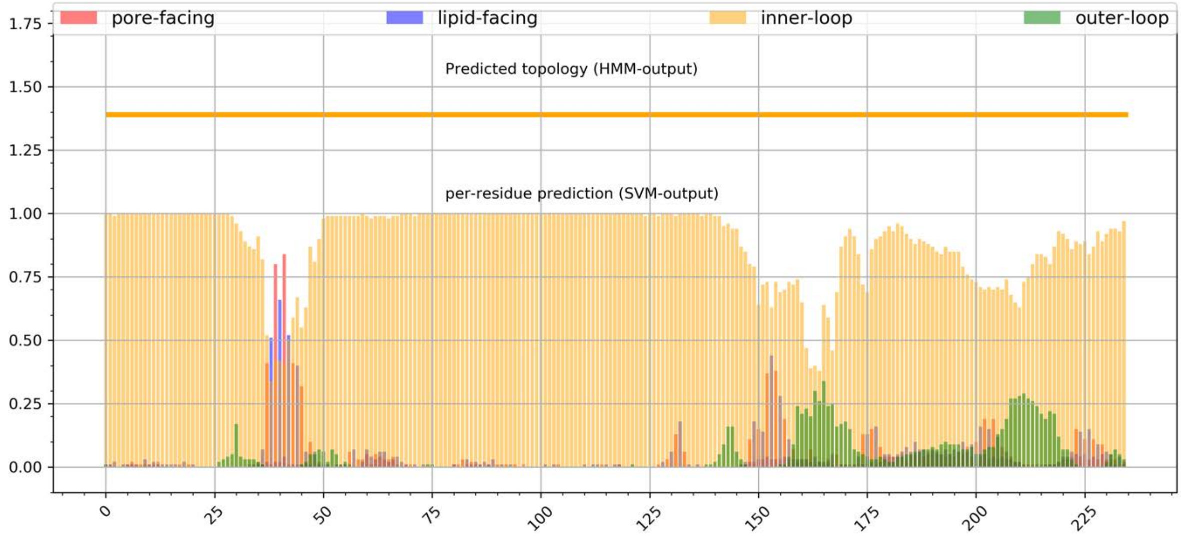

B0V4F6:

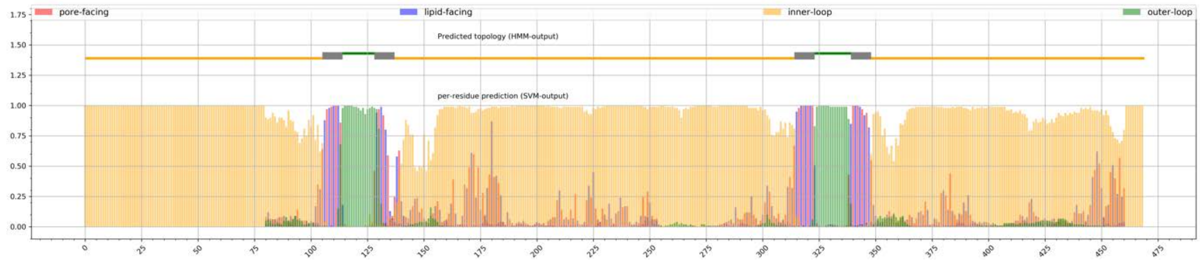

B0V8H0:

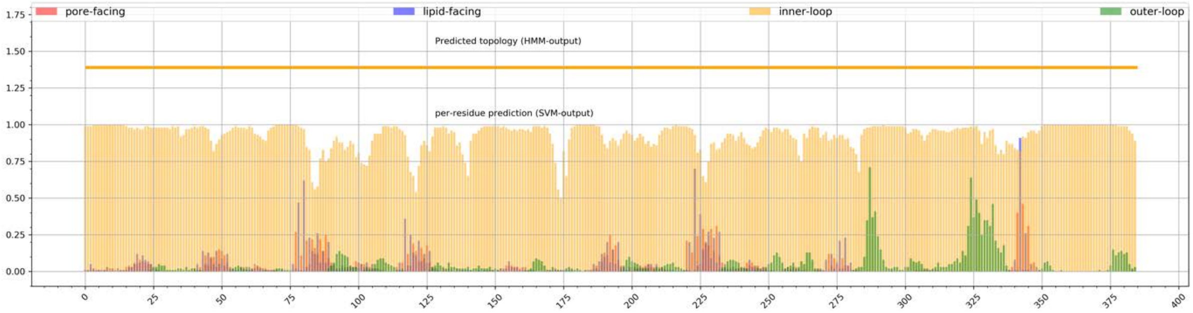

B0VC68:

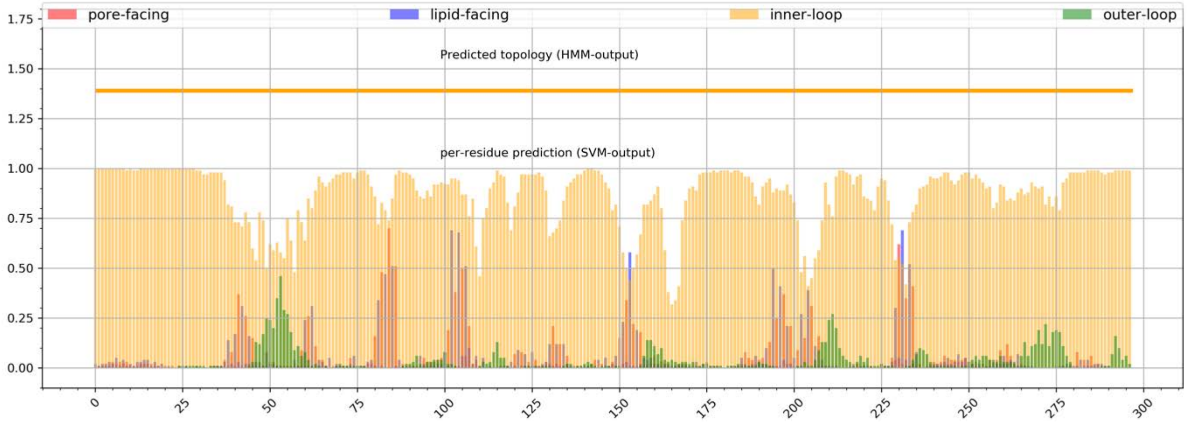

B0VE52:

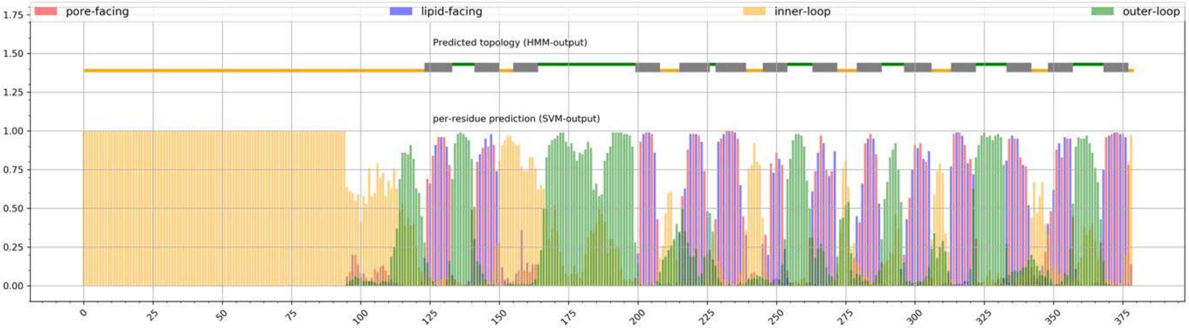

B0V9Z6:

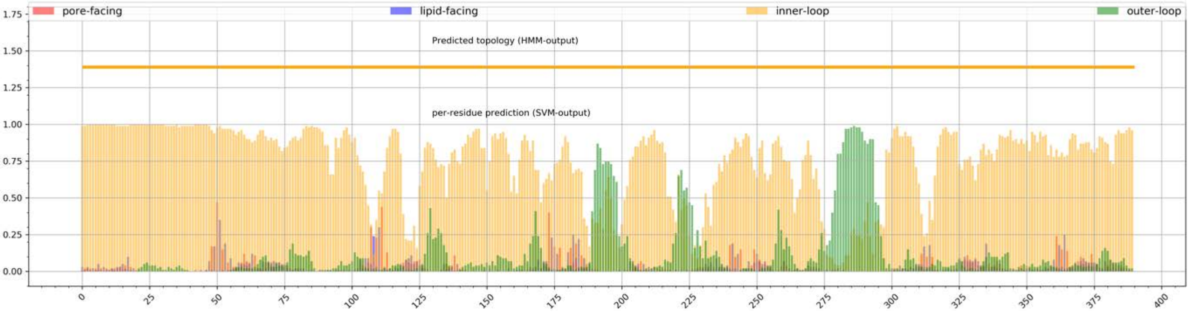

B0VD00:

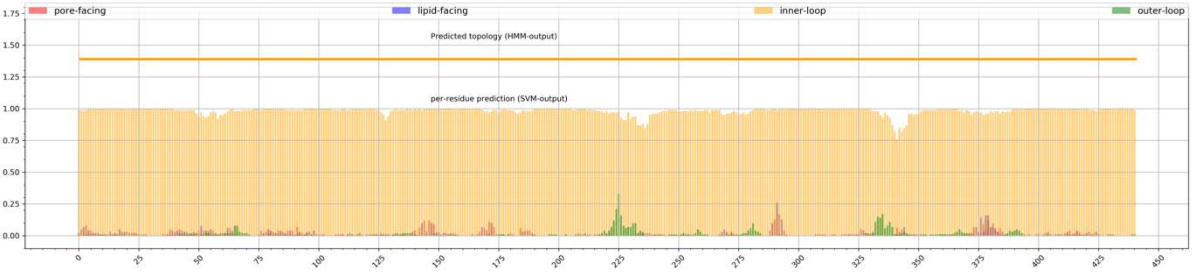

B0V885:

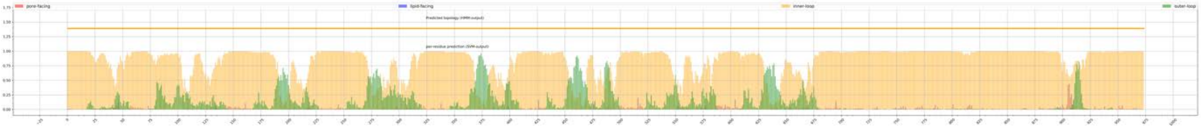

B0VAB5:

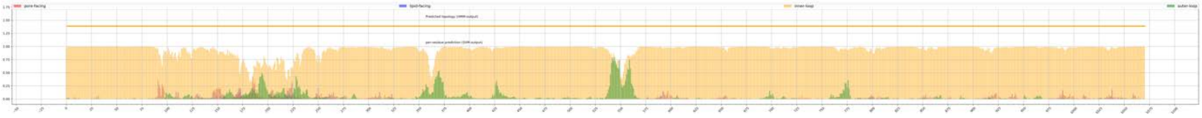

Supplementary Figure 2. Improved topology prediction of the transmembrane beta-barrel structure of the candidate proteins was depicted by BOCTOPUS 2. Default parameters were used the beta barrel structure of selected proteins were depicted under accession numbers.

TMHMM posterior probabilities Results

A0A0R4J8Q3:

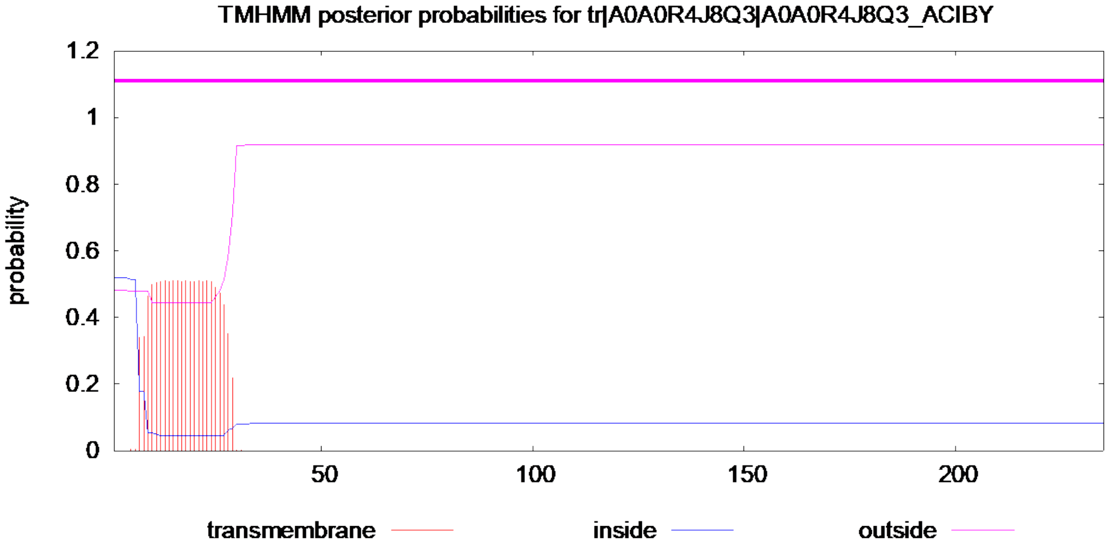

B0V4F6:

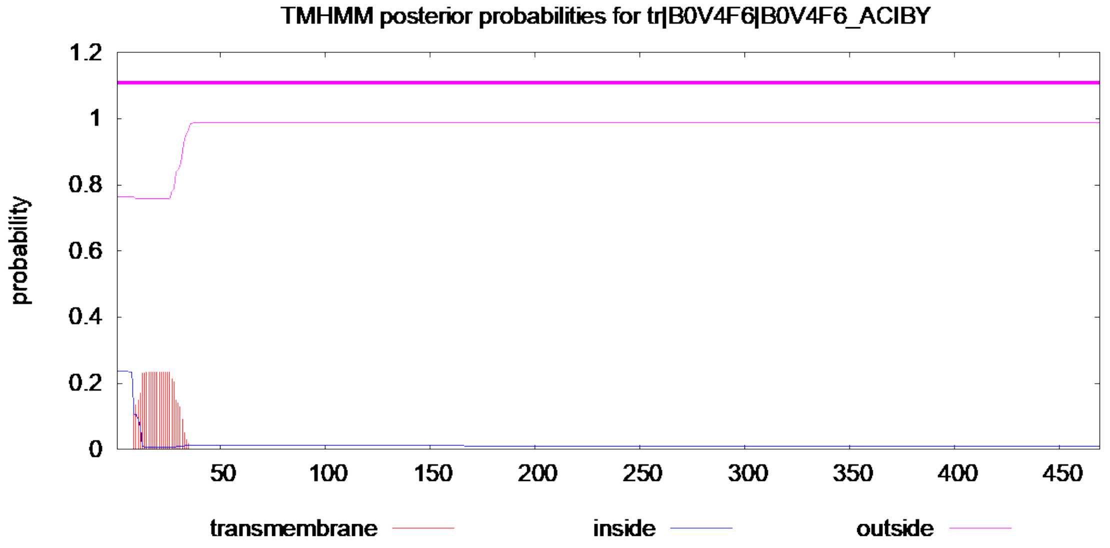

B0V8H0:

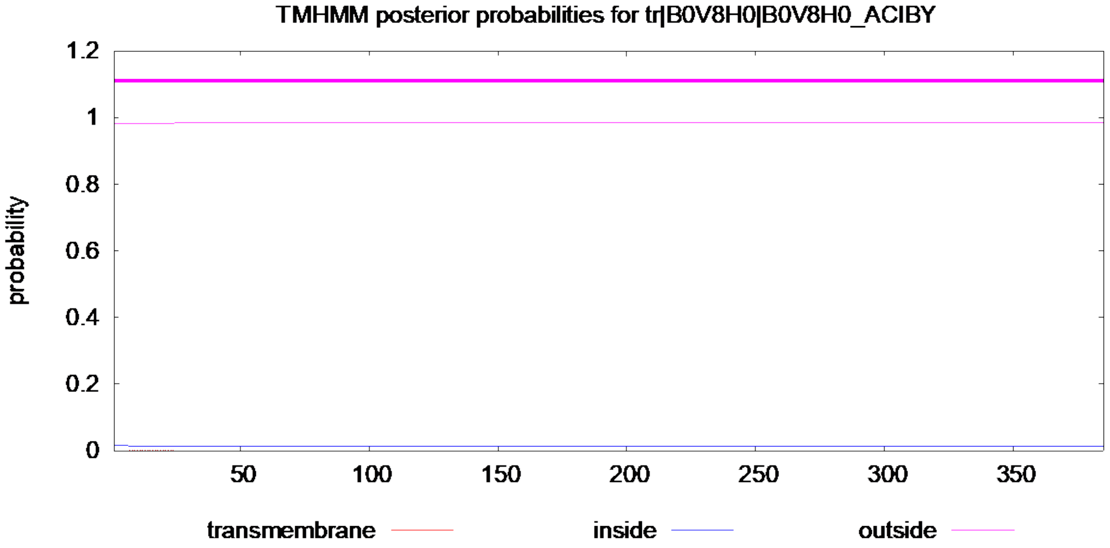

B0VC68:

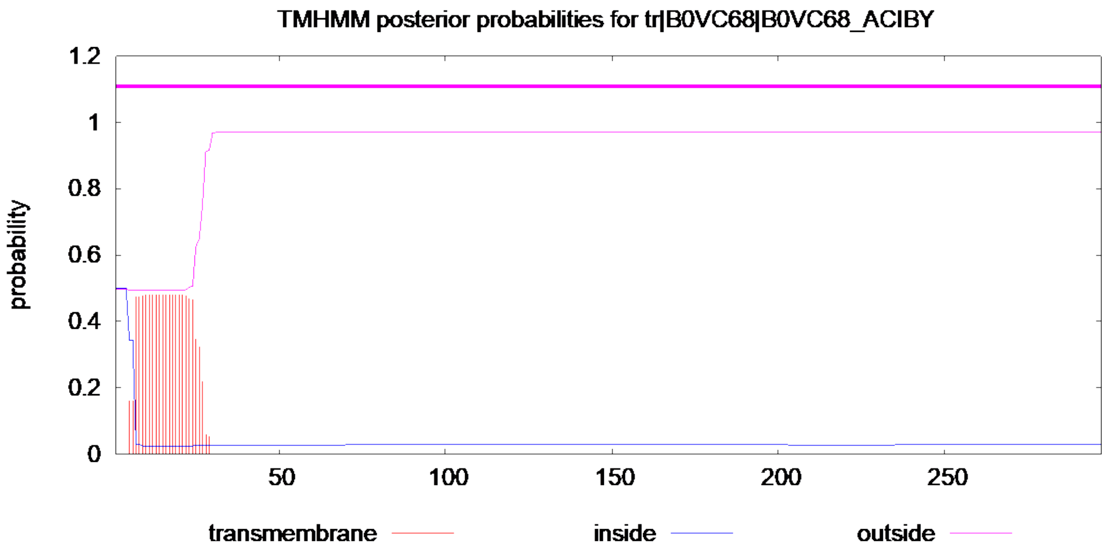

B0VE52:

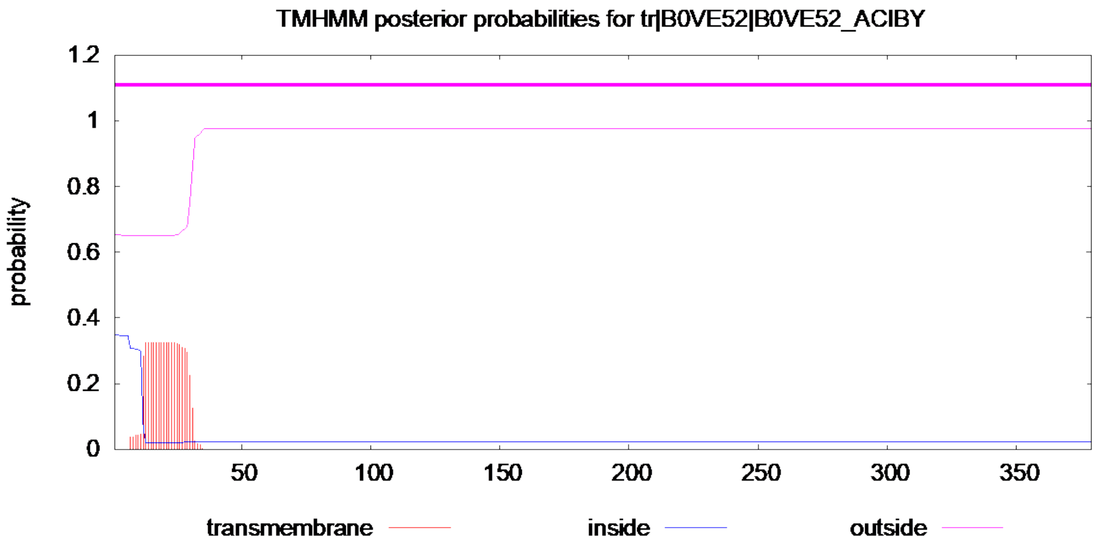

B0V9Z6:

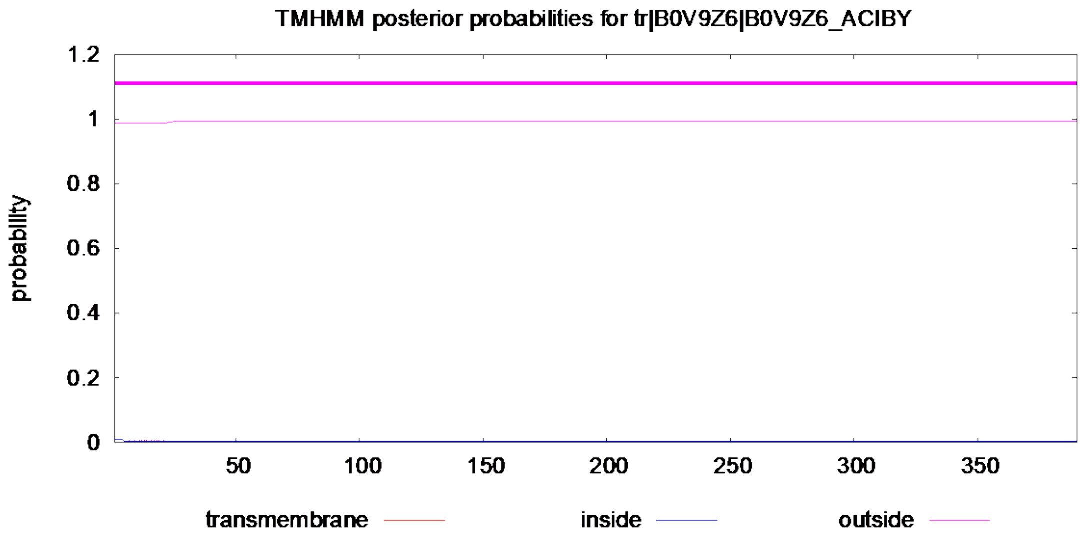

B0VD00:

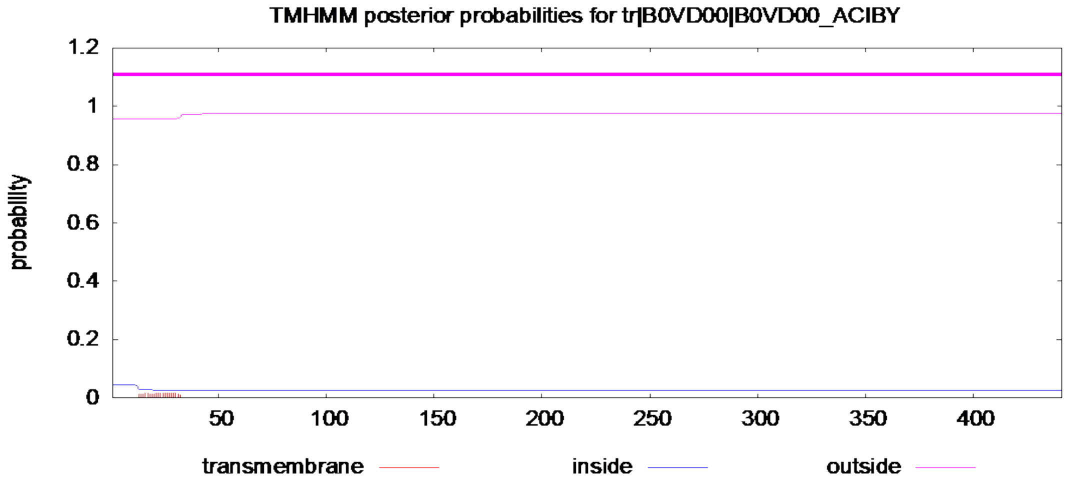

B0V885:

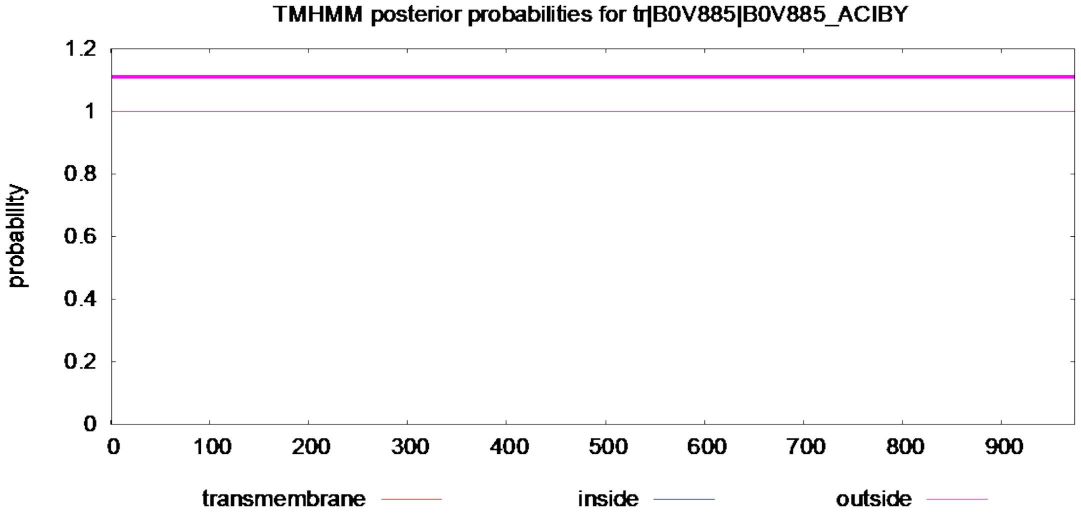

B0VAB5:

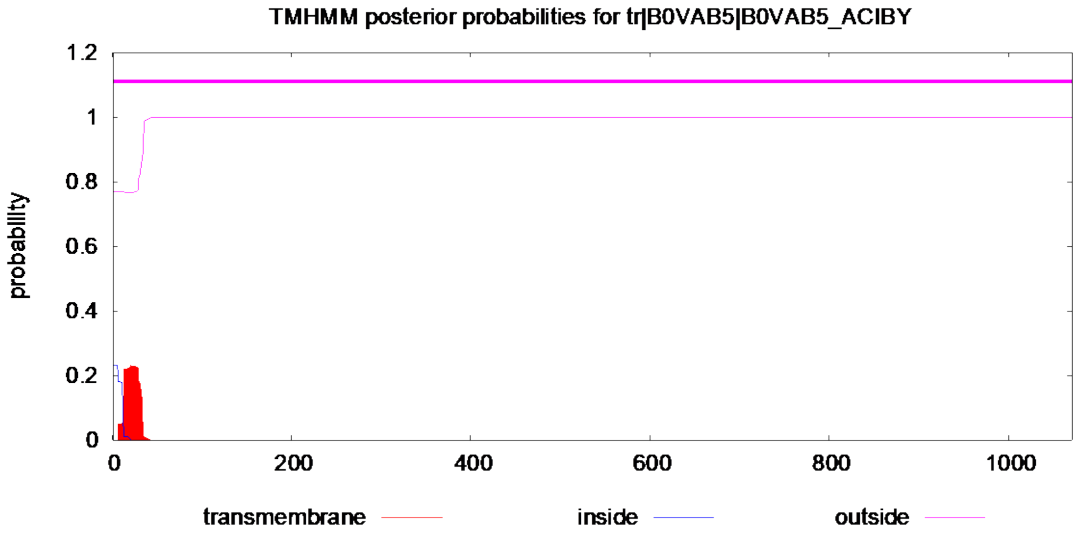

Supplementary Figure 3. TMHMM posterior probabilities results of nine selected proteins. Default parameters were used, and the outputs of analysis were gathered within the graphics.

Supplementary Table 2: Solubility scores of identified 34 proteins were determined via PROSO II. Default parameters were used, the solubility threshold value was set to be 0.6.

Table 2: PROSO Scores of Identified Proteins

Accession:	Result:	PROSO II Score:
B0VDQ8	insoluble	0.345
B0V8N9	insoluble	0.479
B0V5K3	insoluble	0.272
B0V7B6	insoluble	0.452
B0VAQ2	insoluble	0.265
B0VDD7	insoluble	0.551
B0VAS1	insoluble	0.495
B0VEL2	insoluble	0.552
B0VDM9	insoluble	0.460
B0V9C5	insoluble	0.306
B0VEP7	insoluble	0.409
A7FB56	insoluble	0.350
A3M7F6	insoluble	0.198
B0V885	insoluble	0.472
B0VCX9	insoluble	0.525
B0VEM1	insoluble	0.306
B0V4Y0	insoluble	0.469
B0V5U8	insoluble	0.576
B0VB25	insoluble	0.481
B0VCP7	insoluble	0.316
B0V6D8	insoluble	0.382
B0V9U0	insoluble	0.401
B0V6Z4	insoluble	0.257
B0VCS2	insoluble	0.318
B0V4C4	insoluble	0.280
B0V706	insoluble	0.314
B0VDH2	insoluble	0.092
B0V7B7	soluble	0.664
B0VC68	soluble	0.737
B0V4F6	soluble	0.645
B0VAB5	soluble	0.604
B0V9Z6	soluble	0.719
B0VE52	soluble	0.624
B0VD00	soluble	0.602
B0V8H0	soluble	0.659
A0A0R4J8Q3	soluble	0.814
B0V885*	soluble	0.647

* Epitope region of proteins

Supplementary Table 3. Potential O-Linked glycosylated sites of candidate proteins were predicted via GlycoPP. Predictions based on Binary Profile of Patterns and SVM (Support Vector Machine) threshold were used as default.

GlycoPP Results

A0A0R4J8Q3:

>tr_A0A0R4J8Q3_A0A0R4J8Q3_A   Length = 235

Potential O-Linked Glycosylated Sites:

MENDMKKISLVIAASTMSLSVFAAAPITNKSPAKDQFSYSYGYLMGRNNTDALTDLNLDIFY
QGLQEGAQNKTARLTDEEMAKAINDYKKTLEAKQLVEF

QKQGQQNAQAGAAFLAENAKKSGVVTTKSGLQYQVLKEGSGKTPKATSRVKVNYEGRLL
DGTVFDSSIARNHPVDFQLNQVIAGWTEGLQTMKEGGKTR

FFIPAKLAYGEVGAGDSIGPNSTLIFDIELLQVLPK

Position	Residue	Score	Prediction
9	S	-0.33616954	Non-glycosylated
**15**	S	**0.80123845**	**Potential Glycosylated**
16	T	-0.45153503	Non-glycosylated
18	S	-0.1944752	Non-glycosylated
**20**	S	**0.38663001**	**Potential Glycosylated**
**28**	T	**0.58851779**	**Potential Glycosylated**
**31**	S	**0.26381689**	**Potential Glycosylated**
38	S	-0.11148611	Non-glycosylated
**40**	S	**0.25410839**	**Potential Glycosylated**
50	T	-0.42948743	Non-glycosylated
54	T	-1.1308928	Non-glycosylated
**73**	T	**0.26521192**	**Potential Glycosylated**
77	T	-1.2245187	Non-glycosylated
91	T	-0.043575964	Non-glycosylated

**122**	S	**0.045435053**	**Potential Glycosylated**
**126**	T	**0.19906368**	**Potential Glycosylated**
**127**	T	**0.24216799**	**Potential Glycosylated**
129	S	-0.086994803	Non-glycosylated
**140**	S	**0.41637138**	**Potential Glycosylated**
143	T	-1.2643878	Non-glycosylated
147	T	-0.54980353	Non-glycosylated
**148**	S	**0.11989609**	**Potential Glycosylated**
162	T	-0.57133568	Non-glycosylated
**166**	S	**0.089544578**	**Potential Glycosylated**
167	S	-0.24086714	Non-glycosylated
186	T	-0.24023368	Non-glycosylated
**191**	T	**0.067684286**	**Potential Glycosylated**
**198**	T	**0.60445027**	**Potential Glycosylated**
**216**	S	**1.5242939**	**Potential Glycosylated**
**221**	S	**0.57929956**	**Potential Glycosylated**
222	T	-0.18495397	Non-glycosylated

****

B0V4F6:

>tr_B0V4F6_B0V4F6_ACIBYOut Length = 469

Potential O-Linked Glycosylated Sites:

MENTMSKSAIVSRGLILSTLSVTLVACVNMQAPQPAITSHIPQNFSQNHSGKTIAEKSYKEFI
SDPKLVQVIEISLNNNRDLRTATLNIERVQQQYQITK

NSQLPTIGVTGNAVRQVSPSINPNNPVSTFQVGLGMTAYELDFWGRVQNLKDAALNNYLAT
QSAKEAVQISLISNITQVWLNYAFAQANLNLAEQTLKA

QVDAYNLNKKRFDVGIDSEVPLKQAQISVETARNDVATYKTQIQQAKNLLDLLAGHPVPQNL
LPNHAIQNITFEKNFAAGLPSDLLNHRPDLKAAEYEL

RAAGANIGAAKARMFPTISLTGSTGYASSELKDLFKTGNFAWSIGPNIDLPIFDWGTRKTNIK
IAETDQKIALAKYEKAIQSAFREVNDALATHAHIGE

RLDAQRRLVSATAATYKLSMARYRAGVDSYFTVLDAQRSAYAAQQGLLALEQMELNNQIEL
YKVLGGGISKV

Position	Residue	Score	Prediction
4	T	-0.27444305	Non-glycosylated
6	S	-0.30909069	Non-glycosylated
**8**	S	**0.80942251**	**Potential Glycosylated**
**12**	S	**0.7807107**	**Potential Glycosylated**
**18**	S	**0.74452594**	**Potential Glycosylated**
19	T	-1.7955562	Non-glycosylated
21	S	-0.59119956	Non-glycosylated
23	T	-0.027100404	Non-glycosylated
**38**	T	**0.25560482**	**Potential Glycosylated**
**39**	S	**0.65689556**	**Potential Glycosylated**
46	S	-0.00099328701	Non-glycosylated
**50**	S	**0.35263786**	**Potential Glycosylated**
**53**	T	**0.14920887**	**Potential Glycosylated**
58	S	-0.33408557	Non-glycosylated
64	S	-0.31877273	Non-glycosylated
75	S	-0.31013546	Non-glycosylated
84	T	-1.5028375	Non-glycosylated
86	T	-0.19001143	Non-glycosylated
99	T	-0.72019624	Non-glycosylated
102	S	-0.064094053	Non-glycosylated
106	T	-0.66145659	Non-glycosylated
**110**	T	**0.46537988**	**Potential Glycosylated**
**118**	S	**0.52555471**	**Potential Glycosylated**

**120**	S	**0.66367102**	**Potential Glycosylated**
**128**	S	**0.58747274**	**Potential Glycosylated**
**129**	T	**0.10887058**	**Potential Glycosylated**
137	T	-0.40407965	Non-glycosylated
161	T	-0.27405135	Non-glycosylated
163	S	-0.35405272	Non-glycosylated
171	S	-0.12583755	Non-glycosylated
174	S	-0.29158332	Non-glycosylated
177	T	-0.63248336	Non-glycosylated
196	T	-1.055986	Non-glycosylated
**217**	S	**0.47405153**	**Potential Glycosylated**
**227**	S	**0.10488281**	**Potential Glycosylated**
230	T	-0.56663542	Non-glycosylated
237	T	-0.36664284	Non-glycosylated
**240**	T	**0.12022324**	**Potential Glycosylated**
271	T	-0.58601952	Non-glycosylated
282	S	-0.51321023	Non-glycosylated
**315**	T	**0.29821508**	**Potential Glycosylated**
317	S	-0.092992964	Non-glycosylated
**319**	T	**0.44684391**	**Potential Glycosylated**
**321**	S	**0.34880573**	**Potential Glycosylated**
**322**	T	**0.42953712**	**Potential Glycosylated**
326	S	-0.28541686	Non-glycosylated
**327**	S	**0.20359224**	**Potential Glycosylated**
335	T	-0.19718777	Non-glycosylated
**341**	S	**0.61061702**	**Potential Glycosylated**
355	T	-1.0044627	Non-glycosylated

358	T	-0.86749726	Non-glycosylated
365	T	-1.3623689	Non-glycosylated
380	S	-0.36337121	Non-glycosylated
391	T	-0.7085875	Non-glycosylated
**407**	S	**0.10049847**	**Potential Glycosylated**
**409**	T	**0.28449092**	**Potential Glycosylated**
**412**	T	**0.03566691**	**Potential Glycosylated**
**416**	S	**0.10593782**	**Potential Glycosylated**
**426**	S	**0.75105793**	**Potential Glycosylated**
429	T	-0.35874882	Non-glycosylated
**436**	S	**0.66763851**	**Potential Glycosylated**
**467**	S	**0.47818399**	**Potential Glycosylated**

****

B0V8H0:

>tr_B0V8H0_B0V8H0_ACIBYOut Length = 385

Potential O-Linked Glycosylated Sites:

MSLPRYKITMLALSLGVASAFVGCSSNPSKKEVVDTGPQSSEQAYFDKAQKALDRGQYLD
ATKSLEAIDTYYPTGQYAQQAQLELLYSKFKQKDYEGAIA

LAERFIRLNPQHPNVDYAYYVRGVSNMEMNYDSLLRYTSLQQSHRDVSYLKVAYQNFVDLI
RRFPSSQYSVDAAQRMKFIGQELAESEMNAARFNVKRK

AWIAAAERSQWVIEHYPQTPQVPEALATLAYSYDQLGDKATSQQYIEVLKLNYPSLVNKNG
TVNMRAARKEGNWINRATLGILGRESKSVTPDTTTSSE

AEPKRSLLNRVSFGLIGNSGKEETEETTNTPVEAPKSERSWTNRLSFGLLDKPEPKAAEGA
TIAPATSSSEAPSASPADNEADDAAQ

Position	Residue	Score	Prediction
2	S	-0.62871336	Non-glycosylated
9	T	-0.090121392	Non-glycosylated

**14**	S	**0.75034037**	**Potential Glycosylated**
**19**	S	**1.5538959**	**Potential Glycosylated**
**25**	S	**0.39552134**	**Potential Glycosylated**
26	S	-0.001032318	Non-glycosylated
29	S	-0.31986502	Non-glycosylated
**36**	T	**0.32669068**	**Potential Glycosylated**
**40**	S	**0.062289157**	**Potential Glycosylated**
41	S	-0.58267853	Non-glycosylated
62	T	-1.7724051	Non-glycosylated
64	S	-0.45260193	Non-glycosylated
**70**	T	**0.089721683**	**Potential Glycosylated**
74	T	-0.036755205	Non-glycosylated
88	S	-1.060815	Non-glycosylated
125	S	-0.56038224	Non-glycosylated
**133**	S	**0.33224972**	**Potential Glycosylated**
138	T	-0.48138696	Non-glycosylated
**139**	S	**0.12413811**	**Potential Glycosylated**
143	S	-0.16460752	Non-glycosylated
148	S	-0.19683764	Non-glycosylated
166	S	-0.56146214	Non-glycosylated
167	S	-0.070390872	Non-glycosylated
**170**	S	**0.48824218**	**Potential Glycosylated**
187	S	-0.59878797	Non-glycosylated
**208**	S	**0.2668566**	**Potential Glycosylated**
218	T	-0.17950109	Non-glycosylated
227	T	-0.64786398	Non-glycosylated
**231**	S	**0.31723007**	**Potential Glycosylated**

240	T	-0.88477573	Non-glycosylated
**241**	S	**0.47349056**	**Potential Glycosylated**
254	S	-0.1235219	Non-glycosylated
261	T	-0.71741456	Non-glycosylated
278	T	-1.085348	Non-glycosylated
286	S	-0.49644091	Non-glycosylated
288	S	-0.34552202	Non-glycosylated
**290**	T	**0.33724053**	**Potential Glycosylated**
**293**	T	**0.69195931**	**Potential Glycosylated**
294	T	-0.34083901	Non-glycosylated
295	T	-0.50810493	Non-glycosylated
**296**	S	**0.075029311**	**Potential Glycosylated**
297	S	-0.38326039	Non-glycosylated
**304**	S	**0.18174411**	**Potential Glycosylated**
**310**	S	**0.39733222**	**Potential Glycosylated**
317	S	-0.040315553	Non-glycosylated
322	T	-0.47786965	Non-glycosylated
325	T	-0.61526388	Non-glycosylated
326	T	-0.71736228	Non-glycosylated
328	T	-0.43366454	Non-glycosylated
**335**	S	**0.23341807**	**Potential Glycosylated**
338	S	-0.67267448	Non-glycosylated
340	T	-0.2525941	Non-glycosylated
344	S	-0.22576512	Non-glycosylated
**360**	T	**0.69338057**	**Potential Glycosylated**
365	T	-0.34662768	Non-glycosylated
**366**	S	**0.51726062**	**Potential Glycosylated**
**367**	S	**1.3636294**	**Potential Glycosylated**

**368**	S	**0.5961249**	**Potential Glycosylated**
**372**	S	**0.9836512**	**Potential Glycosylated**
**374**	S	**0.27172859**	**Potential Glycosylated**

****

B0VC68:

>tr_B0VC68_B0VC68_ACIBYGlu Length = 297

Potential O-Linked Glycosylated Sites:

MKRSATSLIVSTVMLCTVGATSQIQAADTLAKIKSTGKIVIGHRESSDPISYVVAGKPVGYAV
DICNQFANDIKKELKMPGLKVEYKAVTSSTRIPELLA

GNIDMECGTTTNSKQRQQQVGFSTNYYATEVRMAVKANSGIKSLADLNGKAVVTTQGTTS
DKYIKMNEKGQAINVQNIYGKDHADSFAMMASGRAAAFV

MDDNILAGLIAKSSTPKAFAIVGPVLSSEPYGIMIAKDDPKFKAIADRTVNNLWKTGQMDALY
KKWFLSPIPPKNTNLNMQQSSSYKKLKAHPTDAGI

Position	Residue	Score	Prediction
**4**	S	**0.73199919**	**Potential Glycosylated**
6	T	-0.42975679	Non-glycosylated
**7**	S	**0.55466207**	**Potential Glycosylated**
**11**	S	**1.1933192**	**Potential Glycosylated**
12	T	-0.26730412	Non-glycosylated
**17**	T	**0.46983751**	**Potential Glycosylated**
21	T	-0.27256927	Non-glycosylated
**22**	S	**1.2368629**	**Potential Glycosylated**
**29**	T	**0.45093414**	**Potential Glycosylated**
**35**	S	**0.47055662**	**Potential Glycosylated**
**36**	T	**0.10268522**	**Potential Glycosylated**
**46**	S	**0.69769863**	**Potential Glycosylated**

47	S	-0.0018550017	Non-glycosylated
**51**	S	**0.95828373**	**Potential Glycosylated**
**90**	T	**0.52115751**	**Potential Glycosylated**
**91**	S	**1.0084689**	**Potential Glycosylated**
92	S	-0.17538407	Non-glycosylated
93	T	-0.57632884	Non-glycosylated
**109**	T	**0.021425924**	**Potential Glycosylated**
110	T	-0.52764647	Non-glycosylated
**111**	T	**0.31091239**	**Potential Glycosylated**
**113**	S	**0.022008201**	**Potential Glycosylated**
123	S	-0.11334458	Non-glycosylated
124	T	-0.053981013	Non-glycosylated
129	T	-0.56881642	Non-glycosylated
**139**	S	**0.91302516**	**Potential Glycosylated**
143	S	-0.10612215	Non-glycosylated
154	T	-0.30115433	Non-glycosylated
155	T	-0.18963728	Non-glycosylated
158	T	-0.30130578	Non-glycosylated
**159**	T	**0.36368845**	**Potential Glycosylated**
**160**	S	**0.40486496**	**Potential Glycosylated**
**186**	S	**1.2436255**	**Potential Glycosylated**
**192**	S	**0.58241086**	**Potential Glycosylated**
**212**	S	**0.28045795**	**Potential Glycosylated**
**213**	S	**0.18045484**	**Potential Glycosylated**
**214**	T	**0.25118352**	**Potential Glycosylated**
**226**	S	**0.54481458**	**Potential Glycosylated**
227	S	-0.49290017	Non-glycosylated
248	T	-1.0421755	Non-glycosylated

255	T	-0.75473298	Non-glycosylated
268	S	-0.25448209	Non-glycosylated
275	T	-0.16656891	Non-glycosylated
282	S	-0.23646923	Non-glycosylated
**283**	S	**0.20040875**	**Potential Glycosylated**
284	S	-0.30378745	Non-glycosylated
293	T	-0.1039646	Non-glycosylated

****

B0VE52:

>tr_B0VE52_B0VE52_ACIBYUnc Length = 379

Potential O-Linked Glycosylated Sites:

MKTFHIITYRQLFALLTLGFGSVCMAHAEVNSSSTQQVNGLSSGAQTEENKDENLLDQIPR
WIDATPTIFPEQSNEPIVPPTEQTEDQTWFDRKQKKIRN

WADRTSGKIDNWFGEVDPQKPASATIRVMIDNYWNEYDNYEIKPRIRGKIKLPTLEKRLSVV
FGDDSLDDEFNNSPANINQNPNQDPNKKLDGKRTRDD

NSSIALRWSNFSKKLPFETDADLGIRSGDDIYVRLKASRDWQLRNDFKFYAEQIYRYGIDSE
NYLRTNLELTHARPNQPILSNQFSLTYADDQDDDLTW

ENRLFREHSFFANNRFNYGIYTGGYYNDNDLRLNSWGPFVSWRQPVLREWFFVQGDLNY
FNDHREDRNHYVSTFLRLEALF

Position	Residue	Score	Prediction
3	T	-0.4407511	Non-glycosylated
**8**	T	**0.19749385**	**Potential Glycosylated**
**17**	T	**0.067795475**	**Potential Glycosylated**
**22**	S	**0.74378888**	**Potential Glycosylated**
**32**	S	**0.84112149**	**Potential Glycosylated**
33	S	-0.32993036	Non-glycosylated

**34**	S	**0.58749234**	**Potential Glycosylated**
**35**	T	**0.57688184**	**Potential Glycosylated**
**42**	S	**0.9255043**	**Potential Glycosylated**
43	S	-0.067722345	Non-glycosylated
**47**	T	**0.026413934**	**Potential Glycosylated**
66	T	-0.56481981	Non-glycosylated
**68**	T	**0.4580239**	**Potential Glycosylated**
**74**	S	**0.18923458**	**Potential Glycosylated**
82	T	-0.56969664	Non-glycosylated
85	T	-0.58199553	Non-glycosylated
89	T	-1.0145731	Non-glycosylated
105	T	-0.91680004	Non-glycosylated
106	S	-0.11153012	Non-glycosylated
123	S	-0.081603783	Non-glycosylated
125	T	-0.73873416	Non-glycosylated
154	T	-0.67024181	Non-glycosylated
160	S	-0.60509308	Non-glycosylated
167	S	-0.0011301178	Non-glycosylated
175	S	-0.47733919	Non-glycosylated
196	T	-0.44044818	Non-glycosylated
**201**	S	**0.31271004**	**Potential Glycosylated**
202	S	-0.019817243	Non-glycosylated
208	S	-0.2499095	Non-glycosylated
211	S	-0.023373455	Non-glycosylated
218	T	-0.36967352	Non-glycosylated
**226**	S	**0.9936815**	**Potential Glycosylated**
237	S	-0.33780729	Non-glycosylated
**260**	S	**0.41541036**	**Potential Glycosylated**

266	T	-1.241867	Non-glycosylated
271	T	-1.0620464	Non-glycosylated
**281**	S	**0.22513271**	**Potential Glycosylated**
285	S	-0.89864461	Non-glycosylated
287	T	-1.0584081	Non-glycosylated
297	T	-1.7011434	Non-glycosylated
307	S	-0.28954758	Non-glycosylated
320	T	-0.29526635	Non-glycosylated
333	S	-0.25238723	Non-glycosylated
**339**	S	**0.32796102**	**Potential Glycosylated**
**370**	S	**0.26950267**	**Potential Glycosylated**
371	T	-0.90021131	Non-glycosylated

****

B0V9Z6:

>tr_B0V9Z6_B0V9Z6_ACIBYPut Length = 390

Potential O-Linked Glycosylated Sites:

MMRRLAAPLLCSSFFLLMACGSNNTNSKSPEQNTTAKTEQTNAAKQIYQVEAVAHFKEPW
AITSLPDQRLLVTERQGKLKIFNPQNKQMLDIQGIPAVSY

GGQGGLGDVALHPDFAKNHWIYLSYAAKGQGGSGAVISRAKLDLSNPNQPKLTDVKQIWQ
QVPKVSGQGHYGHRMLFGADGKLWVSSGERQKFDPAQNM

KSNLGKILRLNDDGSAALGNPFYKQGGVTAEIWSLGHRNPLGMAFDRKGQLWVVEMGPK
GGDELNIITKGENYGYPIVSNGDHYSGQPIPDHHTRPEFK

APEIDWTPVISPSSLIIYRGQQFPAWQNKALIGGLSSEAIIVVDLEHKPVKEVQRLEMKQRIR
GLHEAQDGSIWVIEDGSKARLLKLSKKLS

Position	Residue	Score	Prediction
12	S	-0.25232352	Non-glycosylated

13	S	-0.49554211	Non-glycosylated
22	S	-0.18508732	Non-glycosylated
25	T	-0.55967605	Non-glycosylated
**27**	S	**0.58524079**	**Potential Glycosylated**
29	S	-1.058362	Non-glycosylated
**34**	T	**0.22865138**	**Potential Glycosylated**
**35**	T	**0.7220704**	**Potential Glycosylated**
38	T	-0.30181699	Non-glycosylated
41	T	-0.020637405	Non-glycosylated
**63**	T	**0.13328489**	**Potential Glycosylated**
**64**	S	**0.22597846**	**Potential Glycosylated**
73	T	-1.2316765	Non-glycosylated
**99**	S	**1.0528253**	**Potential Glycosylated**
124	S	-0.27138273	Non-glycosylated
**133**	S	**0.1879238**	**Potential Glycosylated**
**138**	S	**0.57261295**	**Potential Glycosylated**
145	S	-0.7843509	Non-glycosylated
153	T	-0.32610154	Non-glycosylated
**166**	S	**0.79098725**	**Potential Glycosylated**
**186**	S	**0.45121957**	**Potential Glycosylated**
**187**	S	**0.76750056**	**Potential Glycosylated**
**201**	S	**0.11482152**	**Potential Glycosylated**
214	S	-0.76372349	Non-glycosylated
**228**	T	**0.42832655**	**Potential Glycosylated**
**233**	S	**0.70709107**	**Potential Glycosylated**
267	T	-0.89643326	Non-glycosylated
**278**	S	**0.86160465**	**Potential Glycosylated**
**284**	S	**0.044587102**	**Potential Glycosylated**

293	T	-1.6790077	Non-glycosylated
305	T	-0.064349354	Non-glycosylated
**309**	S	**0.41847365**	**Potential Glycosylated**
**311**	S	**0.016751497**	**Potential Glycosylated**
**312**	S	**0.18691076**	**Potential Glycosylated**
334	S	-0.1820076	Non-glycosylated
**335**	S	**0.33125872**	**Potential Glycosylated**
370	S	-0.18989509	Non-glycosylated
378	S	-0.58409866	Non-glycosylated
**386**	S	**0.39342296**	**Potential Glycosylated**
390	S	-0.40373486	Non-glycosylated

****

B0VD00:

>tr_B0VD00_B0VD00_ACIBYCha Length = 441

Potential O-Linked Glycosylated Sites:

MDTTLMKTKHLKQFFKATTLAVLISSSMHSFAQPTDEVVAIVDNSVILKSDLEQGMAEAAHE
LQAQKKEVPPQQYLQFQVLDQLILRQAQLEQVKKYGIK

PDEKSLNEAVLKVASQSGSKSLEAFQQKLDAIAPGTYENLRSRIAEDLAINRLRQQQVMSRI
KISDQDVDNFLKSPQGQAALGNQAHVIHMRISGDNPQ

EVQNVAKEVRSQLAQSNDLNALKKLSTATVKVEGADMGFRPLSDIPAELAARITPLQDGQT
TDLISVRDGVHVLKLLERKQNEQKALVPQYQTRHILIQ

PSEVVSPENAKQIIDSIYKRLKAGEDFATLAATYSNDTGSARDGGSLGWVTPGMMVPEFDK
KMQEIPVGEISEPFQTQFGWHILQVTDKREKDMTHEYQ

ERMARQILGERQFNTEIDSWLREVRANAYVEIKDPSLDKKNLQK

Position	Residue	Score	Prediction
**3**	T	**0.40465759**	**Potential Glycosylated**

4	T	-1.0871755	Non-glycosylated
8	T	-0.81528258	Non-glycosylated
18	T	-0.37175308	Non-glycosylated
**19**	T	**0.76178997**	**Potential Glycosylated**
**25**	S	**0.28536368**	**Potential Glycosylated**
**26**	S	**1.1637523**	**Potential Glycosylated**
27	S	-0.12448662	Non-glycosylated
**30**	S	**0.24479718**	**Potential Glycosylated**
35	T	-0.70092151	Non-glycosylated
45	S	-0.71442176	Non-glycosylated
50	S	-0.80884188	Non-glycosylated
105	S	-1.0392338	Non-glycosylated
**115**	S	**1.3103303**	**Potential Glycosylated**
**117**	S	**0.19206955**	**Potential Glycosylated**
119	S	-0.59182547	Non-glycosylated
**121**	S	**0.31380223**	**Potential Glycosylated**
136	T	-0.49057511	Non-glycosylated
142	S	-0.73566942	Non-glycosylated
160	S	-0.4928352	Non-glycosylated
165	S	-0.51515338	Non-glycosylated
**175**	S	**0.083766193**	**Potential Glycosylated**
**194**	S	**0.70973781**	**Potential Glycosylated**
**210**	S	**0.90944923**	**Potential Glycosylated**
215	S	-1.0311895	Non-glycosylated
**225**	S	**0.57629606**	**Potential Glycosylated**
226	T	-0.9777051	Non-glycosylated
228	T	-0.10328561	Non-glycosylated
**242**	S	**0.27342768**	**Potential Glycosylated**

253	T	-0.23073458	Non-glycosylated
260	T	-0.34353716	Non-glycosylated
261	T	-0.68303849	Non-glycosylated
265	S	-0.50992265	Non-glycosylated
292	T	-0.44171705	Non-glycosylated
300	S	-0.2675446	Non-glycosylated
**304**	S	**0.51512**	**Potential Glycosylated**
**314**	S	**0.56665276**	**Potential Glycosylated**
327	T	-0.95426451	Non-glycosylated
331	T	-0.3642489	Non-glycosylated
**333**	S	**0.23452267**	**Potential Glycosylated**
**336**	T	**0.26965638**	**Potential Glycosylated**
**338**	S	**1.0237974**	**Potential Glycosylated**
**344**	S	**0.18168625**	**Potential Glycosylated**
**349**	T	**0.051511717**	**Potential Glycosylated**
370	S	-0.020281698	Non-glycosylated
**375**	T	**0.065934529**	**Potential Glycosylated**
385	T	-1.4721907	Non-glycosylated
393	T	-0.84716637	Non-glycosylated
412	T	-0.76633438	Non-glycosylated
**416**	S	**0.59299048**	**Potential Glycosylated**
433	S	-1.0712849	Non-glycosylated

****

B0V885:

>tr_B0V885_B0V885_ACIBYPut Length = 974

Potential O-Linked Glycosylated Sites:

MFIKSILSSITSIIPLPENSNTSSNLGNGSGDGLLNGISSGNGEHNYGIGNGIADDASITAPITIP
LNLSGNSITLIGNSSSSSVNSSPTTTSNNVNDND

VTNNGNGSTIGSGTGNGSGDGLLNGAASGNGEHNYGIGNGIADDASITAPLSIPINLAGNSI
TLIGDSSSSSVNNSATNTSNTVNDNDTTYNGNGSGGG

NGSGDGLLNGIGSGNGEQNYGIGNGIADDASITAPITLPINLSGNSITLIGNSSASSVNSSPTT
TSNTVNDNDTTYNGNGTGDSGVSALGGSGNGSGDG

AGNGIASGNGEHNYGIGNGNGDDVDITAPITGVLNISGNSFTLIGNSSSSSVNTAPTTTSNTV
NDNDTIDNGNSGGTGSGSGNGSGDGLLNGAASGNGE

HNYGIGNGNGDDVDITAPITGVFNFSGNSFSIIGNSSSSSINTAPTTTTNTVNDNDVTDNGN
DGGGLVGGSSGNGSGDGLLNGAASGNGEHNYGIGNGN

GDDADFTFPLTGVLNFSGNSLSGFGSSSSDSVNVAPTTATNTVNDNDTIDNANTGGLGDG
SGNGSGDGLLNGAASGNGEHNYGIGNGNGDDADFTLPFT

GGLNILGNALSGIGGSSTDSINISPTTTSNTVNDNDTTNNGNTSGGVIGSGDSGNGSGDGLL
NGISSGNGEHNYGIGNGNGDDVDVVAPITTPLNVLGN

SFSFIGGEGTGDILGPITGIIGGIGGDGDILSPITGIIGGIGGDGDILSPITGIIGSIGGIGGDLGD
NPLTGIIQSGIDVLQNLESLKTGLINTGIDTI

AGTIIGVFPDAEHPVGDFADLGKLLFETSRDSVNGTLEAISDLAGADLEGASGSITGVIDTLIT
NGSTASTIIQHIVGDDLVTENGGLLGSITTIIGGV

DSGDGGLLGGLDGLISINYGDSDNSNSIDVEDILGNILGSVGSNQGIAVGEPDPTGGSLIHTI
SLNTVNQLTDQLLHALPTV

Position	Residue	Score	Prediction
**5**	S	**0.32508295**	**Potential Glycosylated**
**8**	S	**0.86856194**	**Potential Glycosylated**
**9**	S	**0.0090775033**	**Potential Glycosylated**
**11**	T	**0.093726336**	**Potential Glycosylated**
**12**	S	**0.60668755**	**Potential Glycosylated**
20	S	-0.049876721	Non-glycosylated
22	T	-0.06821504	Non-glycosylated
**23**	S	**0.27008037**	**Potential Glycosylated**
24	S	-0.24816971	Non-glycosylated
**30**	S	**0.85824283**	**Potential Glycosylated**

**39**	S	**0.49179007**	**Potential Glycosylated**
**40**	S	**0.52924671**	**Potential Glycosylated**
58	S	-0.33525434	Non-glycosylated
**60**	T	**0.25262969**	**Potential Glycosylated**
64	T	-1.057143	Non-glycosylated
**70**	S	**0.044167121**	**Potential Glycosylated**
**73**	S	**0.1285657**	**Potential Glycosylated**
**75**	T	**0.60840332**	**Potential Glycosylated**
**80**	S	**1.2270201**	**Potential Glycosylated**
**81**	S	**0.50332032**	**Potential Glycosylated**
**82**	S	**0.54499731**	**Potential Glycosylated**
**83**	S	**0.88860168**	**Potential Glycosylated**
84	S	-0.50415009	Non-glycosylated
**87**	S	**0.7505671**	**Potential Glycosylated**
**88**	S	**0.37289649**	**Potential Glycosylated**
90	T	-0.39116843	Non-glycosylated
**91**	T	**0.05724665**	**Potential Glycosylated**
92	T	-0.67155032	Non-glycosylated
93	S	-0.27179585	Non-glycosylated
102	T	-0.5142942	Non-glycosylated
**108**	S	**0.823164**	**Potential Glycosylated**
**109**	T	**0.1851298**	**Potential Glycosylated**
**112**	S	**0.7882908**	**Potential Glycosylated**
**114**	T	**0.42132029**	**Potential Glycosylated**
**118**	S	**0.89820774**	**Potential Glycosylated**
**128**	S	**0.59566657**	**Potential Glycosylated**
146	S	-0.4326033	Non-glycosylated

**148**	T	**0.3887924**	**Potential Glycosylated**
152	S	-0.69700911	Non-glycosylated
161	S	-0.12617994	Non-glycosylated
**163**	T	**0.67846895**	**Potential Glycosylated**
**168**	S	**1.0765113**	**Potential Glycosylated**
169	S	-0.093647771	Non-glycosylated
170	S	-0.28951403	Non-glycosylated
**171**	S	**0.8386202**	**Potential Glycosylated**
172	S	-0.52567815	Non-glycosylated
**176**	S	**0.24206654**	**Potential Glycosylated**
178	T	-0.76068847	Non-glycosylated
180	T	-1.1951229	Non-glycosylated
181	S	-0.090762433	Non-glycosylated
183	T	-0.69531657	Non-glycosylated
**189**	T	**0.68614926**	**Potential Glycosylated**
190	T	-0.50684348	Non-glycosylated
**196**	S	**0.77327284**	**Potential Glycosylated**
**202**	S	**0.92765452**	**Potential Glycosylated**
**212**	S	**0.78552415**	**Potential Glycosylated**
230	S	-0.30735156	Non-glycosylated
232	T	-0.011290691	Non-glycosylated
236	T	-1.1465486	Non-glycosylated
**242**	S	**0.15942701**	**Potential Glycosylated**
**245**	S	**0.16237151**	**Potential Glycosylated**
**247**	T	**0.60408627**	**Potential Glycosylated**
**252**	S	**1.2011494**	**Potential Glycosylated**
**253**	S	**0.69507462**	**Potential Glycosylated**
**255**	S	**0.9087215**	**Potential Glycosylated**

256	S	-0.33306581	Non-glycosylated
**259**	S	**0.68580015**	**Potential Glycosylated**
**260**	S	**0.32384087**	**Potential Glycosylated**
262	T	-0.22558552	Non-glycosylated
**263**	T	**0.27936039**	**Potential Glycosylated**
264	T	-1.0155952	Non-glycosylated
**265**	S	**0.030741924**	**Potential Glycosylated**
267	T	-0.72722408	Non-glycosylated
**273**	T	**0.18793445**	**Potential Glycosylated**
274	T	-0.80238655	Non-glycosylated
**280**	T	**0.69776774**	**Potential Glycosylated**
**283**	S	**1.2969785**	**Potential Glycosylated**
**286**	S	**1.4388464**	**Potential Glycosylated**
**291**	S	**0.82533039**	**Potential Glycosylated**
**295**	S	**0.81047184**	**Potential Glycosylated**
**305**	S	**0.6564375**	**Potential Glycosylated**
**325**	T	**0.040115675**	**Potential Glycosylated**
329	T	-0.29608099	Non-glycosylated
**335**	S	**0.5734831**	**Potential Glycosylated**
338	S	-0.3098537	Non-glycosylated
**340**	T	**0.43241075**	**Potential Glycosylated**
**345**	S	**0.9548741**	**Potential Glycosylated**
**346**	S	**0.14953902**	**Potential Glycosylated**
**347**	S	**0.30771957**	**Potential Glycosylated**
**348**	S	**0.55602513**	**Potential Glycosylated**
349	S	-0.4856428	Non-glycosylated
**352**	T	**0.4391246**	**Potential Glycosylated**

355	T	-0.12784631	Non-glycosylated
356	T	-0.13035861	Non-glycosylated
357	T	-0.3962077	Non-glycosylated
358	S	-0.3471032	Non-glycosylated
360	T	-0.51397649	Non-glycosylated
**366**	T	**0.34381918**	**Potential Glycosylated**
**372**	S	**0.96256636**	**Potential Glycosylated**
**375**	T	**0.16675621**	**Potential Glycosylated**
**377**	S	**0.69448243**	**Potential Glycosylated**
**379**	S	**0.7571624**	**Potential Glycosylated**
**383**	S	**1.2465417**	**Potential Glycosylated**
**393**	S	**0.59566657**	**Potential Glycosylated**
**413**	T	**0.33094016**	**Potential Glycosylated**
417	T	-0.14168248	Non-glycosylated
**423**	S	**0.66164682**	**Potential Glycosylated**
**426**	S	**0.30650698**	**Potential Glycosylated**
**428**	S	**1.2088617**	**Potential Glycosylated**
**433**	S	**0.93484**	**Potential Glycosylated**
**434**	S	**0.44473445**	**Potential Glycosylated**
435	S	-0.063106155	Non-glycosylated
**436**	S	**0.59922307**	**Potential Glycosylated**
437	S	-0.22480315	Non-glycosylated
**440**	T	**0.34760764**	**Potential Glycosylated**
443	T	-0.25113582	Non-glycosylated
444	T	-0.40589307	Non-glycosylated
445	T	-0.43334938	Non-glycosylated
446	T	-0.34901461	Non-glycosylated
448	T	-0.5632408	Non-glycosylated

455	T	-0.78368861	Non-glycosylated
**468**	S	**0.80820037**	**Potential Glycosylated**
**469**	S	**0.19367448**	**Potential Glycosylated**
**473**	S	**0.75185781**	**Potential Glycosylated**
**483**	S	**0.59566657**	**Potential Glycosylated**
503	T	-0.15259373	Non-glycosylated
507	T	-0.47046487	Non-glycosylated
**513**	S	**0.36756215**	**Potential Glycosylated**
**516**	S	**0.75893645**	**Potential Glycosylated**
**518**	S	**1.0843311**	**Potential Glycosylated**
**522**	S	**0.54739473**	**Potential Glycosylated**
**523**	S	**0.86576612**	**Potential Glycosylated**
**524**	S	**0.71731184**	**Potential Glycosylated**
525	S	-0.066871777	Non-glycosylated
**527**	S	**0.060457887**	**Potential Glycosylated**
533	T	-0.30213602	Non-glycosylated
534	T	-0.57144009	Non-glycosylated
536	T	-1.1584535	Non-glycosylated
538	T	-0.97407622	Non-glycosylated
**544**	T	**0.052270659**	**Potential Glycosylated**
**550**	T	**0.35005733**	**Potential Glycosylated**
**557**	S	**0.652672**	**Potential Glycosylated**
**561**	S	**0.66000597**	**Potential Glycosylated**
**571**	S	**0.59566657**	**Potential Glycosylated**
591	T	-0.21215889	Non-glycosylated
**595**	T	**0.091823192**	**Potential Glycosylated**
**606**	S	**1.223852**	**Potential Glycosylated**

**611**	S	**1.1478898**	**Potential Glycosylated**
**612**	S	**1.2508438**	**Potential Glycosylated**
613	T	-0.24949583	Non-glycosylated
**615**	S	**0.13151956**	**Potential Glycosylated**
**619**	S	**0.30054792**	**Potential Glycosylated**
621	T	-0.4867318	Non-glycosylated
622	T	-0.23977029	Non-glycosylated
623	T	-1.0318582	Non-glycosylated
624	S	-0.25714891	Non-glycosylated
626	T	-0.58050667	Non-glycosylated
**632**	T	**0.21658186**	**Potential Glycosylated**
633	T	-0.5657919	Non-glycosylated
**638**	T	**0.37727272**	**Potential Glycosylated**
**639**	S	**1.039204**	**Potential Glycosylated**
**645**	S	**1.5951766**	**Potential Glycosylated**
**648**	S	**0.4056488**	**Potential Glycosylated**
**652**	S	**0.0820638**	**Potential Glycosylated**
**661**	S	**0.49179007**	**Potential Glycosylated**
**662**	S	**0.52924671**	**Potential Glycosylated**
686	T	-0.62213712	Non-glycosylated
687	T	-0.14483538	Non-glycosylated
**695**	S	**0.74700853**	**Potential Glycosylated**
**697**	S	**0.53576557**	**Potential Glycosylated**
**704**	T	**0.088186582**	**Potential Glycosylated**
**712**	T	**0.86077074**	**Potential Glycosylated**
**726**	S	**0.76707346**	**Potential Glycosylated**
**729**	T	**0.56753477**	**Potential Glycosylated**
**743**	S	**0.76607231**	**Potential Glycosylated**

**746**	T	**0.48802986**	**Potential Glycosylated**
**751**	S	**1.2558534**	**Potential Glycosylated**
**765**	T	**0.18758378**	**Potential Glycosylated**
**770**	S	**0.69740547**	**Potential Glycosylated**
780	S	-0.14099542	Non-glycosylated
783	T	-0.96714381	Non-glycosylated
**788**	T	**0.34723022**	**Potential Glycosylated**
**792**	T	**0.63482934**	**Potential Glycosylated**
**796**	T	**0.16689768**	**Potential Glycosylated**
821	T	-1.2333734	Non-glycosylated
822	S	-0.31029007	Non-glycosylated
825	S	-0.55344808	Non-glycosylated
829	T	-0.46248519	Non-glycosylated
**834**	S	**0.50513002**	**Potential Glycosylated**
**845**	S	**0.64147181**	**Potential Glycosylated**
**847**	S	**0.007024225**	**Potential Glycosylated**
**849**	T	**0.16113917**	**Potential Glycosylated**
854	T	-0.46745192	Non-glycosylated
857	T	-0.25377054	Non-glycosylated
860	S	-0.14249992	Non-glycosylated
**861**	T	**0.0025343363**	**Potential Glycosylated**
**863**	S	**0.4837251**	**Potential Glycosylated**
864	T	-0.13782915	Non-glycosylated
876	T	-1.4819507	Non-glycosylated
884	S	-0.063524512	Non-glycosylated
**886**	T	**0.83870354**	**Potential Glycosylated**
**887**	T	**0.90876983**	**Potential Glycosylated**

**894**	S	**1.4864613**	**Potential Glycosylated**
908	S	-0.198221	Non-glycosylated
914	S	-0.13157664	Non-glycosylated
917	S	-0.59976293	Non-glycosylated
**919**	S	**0.3940876**	**Potential Glycosylated**
**932**	S	**0.13237578**	**Potential Glycosylated**
**935**	S	**0.35310653**	**Potential Glycosylated**
**947**	T	**0.51821429**	**Potential Glycosylated**
950	S	-0.21145894	Non-glycosylated
954	T	-0.02074292	Non-glycosylated
956	S	-0.79718706	Non-glycosylated
959	T	-1.344365	Non-glycosylated
964	T	-0.53126684	Non-glycosylated
973	T	-0.33381334	Non-glycosylated

****

B0VAB5:

>tr_B0VAB5_B0VAB5_ACIBYPut Length = 1071

Potential O-Linked Glycosylated Sites:

MYKPTTFVWQPSAASLFKITVLSSALAALGITTGCSSTPQSAKTSKTKQVSGAGYLDASSLD
SLEDLLSATDMRAVEGDRLLILKHGDVWKRMAVGFKMD

LNHWDPRIEAQRSWFISRQPYLDRLSARASRYLYHTVKEAERRGLPTELALLPVIESSYDPA
ATSSAAAAGLWQFIPSTGRIYGLQQTGMYDGRRDVVE

STRAAYEFLGSLYNQFGSWELALAAYNAGPGRIQQAINRNQAAGLPTDYWSLKLPQETMN
YVPRFLAVAQIIKNPRAYGVSLPPIANRPHFREVTLSAP

LSLNEIASVTGLSRAELYALNPGYRGETVDPASPMRILIPADISPSVDNKLKGMKAGGSSGW
WASVTSPSKPTTTTSTSVTVRTTPSTPAQPVRPSTPA

KTSSSSVTVKTTTPRGSDALAAFAASADVPSAPRIPVAVTPAANIKPVRTEPPISATEREKILA
AVRAEGEKETVDQALEPQATQAEKDQVVAELKALA

PQGTEIVDPYDGKIKLTAIQTSQSVAEQQGKEVSKGFAYPKTLAEDATLANSEDAQRNKDK
PYIKTDTDVVVVQPKGKRSTYTVQPGDTLAVIAMKNGV

NWRDVAKWNQIDPEKTLFVGTSLYLYDAKPQEAETTAKSAAKPDVYVVQANDSLTGVANQ
FNLSVKQLAEYNDLSVTDGLFVGQKLQLKEPKGNRAAKV

EPKAIQASTRRIATKSYTVKRGEYLKLIADRYALSNQELADLTPGLSAGSNLIVGQKINVPAK
EITVDEVDDSKASGKYEKLAAGPSYKTESYKVQRGD

TLSSIATKSKISLAELAELNNLKANSHVQLGQTLKVPAGASVPDQYVVQSGDSLNAIAAKYNL
QTSYLADLNGLSRTAGLRAGQRLKLTGEVETTSKVS

AKNTKEETPETYTVKSGDSLGNIANRYHLQLDYLAALNGLSRNSNVRVGQRLKLTGDLPTV
ETAKTDTAKSSPKAVVAGKNTEKYTVKAGESLNAIASR

AGISVRELAEMNALKANANLQRGQNIVIPKTVVEYKVKRGDTLIGLASKYGLETTLLAELNNL
TPSTQLRIGDIIKVPNL

Position	Residue	Score	Prediction
**5**	T	**0.49609488**	**Potential Glycosylated**
6	T	-0.30363914	Non-glycosylated
**12**	S	**0.42910506**	**Potential Glycosylated**
**15**	S	**0.19816401**	**Potential Glycosylated**
**20**	T	**0.054324165**	**Potential Glycosylated**
23	S	-0.30682029	Non-glycosylated
24	S	-0.64797761	Non-glycosylated
**32**	T	**1.2545241**	**Potential Glycosylated**
**33**	T	**1.0720471**	**Potential Glycosylated**
**36**	S	**0.48816071**	**Potential Glycosylated**
**37**	S	**0.11147536**	**Potential Glycosylated**
38	T	-0.45377688	Non-glycosylated
**41**	S	**0.92261001**	**Potential Glycosylated**
**44**	T	**0.8862066**	**Potential Glycosylated**
45	S	-0.1970565	Non-glycosylated
47	T	-0.064601695	Non-glycosylated
**51**	S	**0.4893136**	**Potential Glycosylated**

59	S	-0.4957127	Non-glycosylated
60	S	-0.16017538	Non-glycosylated
63	S	-0.48487709	Non-glycosylated
69	S	-0.66176765	Non-glycosylated
71	T	-1.0338734	Non-glycosylated
113	S	-0.90951198	Non-glycosylated
**117**	S	**0.28217393**	**Potential Glycosylated**
126	S	-0.27525928	Non-glycosylated
130	S	-1.2269522	Non-glycosylated
136	T	-0.5178035	Non-glycosylated
147	T	-0.5421545	Non-glycosylated
157	S	-0.090872302	Non-glycosylated
**158**	S	**0.66786971**	**Potential Glycosylated**
**164**	T	**0.26163542**	**Potential Glycosylated**
**165**	S	**0.66308188**	**Potential Glycosylated**
**166**	S	**0.73947187**	**Potential Glycosylated**
**178**	S	**0.97812863**	**Potential Glycosylated**
179	T	-0.31728867	Non-glycosylated
188	T	-0.38457468	Non-glycosylated
**200**	S	**0.12501872**	**Potential Glycosylated**
201	T	-1.3882553	Non-glycosylated
**210**	S	**0.11975296**	**Potential Glycosylated**
217	S	-0.43230928	Non-glycosylated
**246**	T	**0.11923987**	**Potential Glycosylated**
250	S	-0.10675486	Non-glycosylated
257	T	-0.51476581	Non-glycosylated
**280**	S	**0.49406995**	**Potential Glycosylated**
**294**	T	**0.2102276**	**Potential Glycosylated**

**296**	S	**0.10474462**	**Potential Glycosylated**
300	S	-0.62667436	Non-glycosylated
**306**	S	**0.042073418**	**Potential Glycosylated**
308	T	-0.22768767	Non-glycosylated
**311**	S	**0.61668592**	**Potential Glycosylated**
326	T	-0.32221442	Non-glycosylated
**331**	S	**0.38796426**	**Potential Glycosylated**
342	S	-0.25223478	Non-glycosylated
344	S	-0.21597685	Non-glycosylated
**357**	S	**0.39631879**	**Potential Glycosylated**
**358**	S	**1.0420124**	**Potential Glycosylated**
**363**	S	**0.25663477**	**Potential Glycosylated**
**365**	T	**0.15296368**	**Potential Glycosylated**
**366**	S	**0.69196141**	**Potential Glycosylated**
**368**	S	**0.49440807**	**Potential Glycosylated**
**371**	T	**0.46029179**	**Potential Glycosylated**
372	T	-0.36951385	Non-glycosylated
**373**	T	**0.86065553**	**Potential Glycosylated**
374	T	-0.37956483	Non-glycosylated
**375**	S	**0.20568461**	**Potential Glycosylated**
**376**	T	**0.41958089**	**Potential Glycosylated**
377	S	-0.17582355	Non-glycosylated
**379**	T	**0.56268066**	**Potential Glycosylated**
**382**	T	**0.40982122**	**Potential Glycosylated**
383	T	-0.048375795	Non-glycosylated
**385**	S	**0.65148116**	**Potential Glycosylated**
386	T	-0.010160152	Non-glycosylated

394	S	-0.059736842	Non-glycosylated
**395**	T	**0.59800318**	**Potential Glycosylated**
**399**	T	**0.32981702**	**Potential Glycosylated**
**400**	S	**0.32084817**	**Potential Glycosylated**
401	S	-0.04638807	Non-glycosylated
**402**	S	**0.72124687**	**Potential Glycosylated**
403	S	-0.56729019	Non-glycosylated
**405**	T	**0.12266283**	**Potential Glycosylated**
**408**	T	**0.45141312**	**Potential Glycosylated**
**409**	T	**0.26407647**	**Potential Glycosylated**
**410**	T	**0.80457887**	**Potential Glycosylated**
414	S	-0.35083332	Non-glycosylated
**423**	S	**1.0631098**	**Potential Glycosylated**
**428**	S	**0.21089226**	**Potential Glycosylated**
437	T	-0.62403247	Non-glycosylated
447	T	-0.095341845	Non-glycosylated
**452**	S	**0.60758746**	**Potential Glycosylated**
454	T	-0.87766655	Non-glycosylated
471	T	-0.34700518	Non-glycosylated
481	T	-0.67700939	Non-glycosylated
500	T	-0.62793553	Non-glycosylated
513	T	-0.047285687	Non-glycosylated
517	T	-0.4522664	Non-glycosylated
**518**	S	**0.86007242**	**Potential Glycosylated**
520	S	-0.038833569	Non-glycosylated
**530**	S	**0.3318803**	**Potential Glycosylated**
538	T	-0.66749528	Non-glycosylated
544	T	-0.96564257	Non-glycosylated

**548**	S	**0.095716693**	**Potential Glycosylated**
562	T	-0.65256103	Non-glycosylated
564	T	-0.96479638	Non-glycosylated
576	S	-0.051424325	Non-glycosylated
577	T	-1.0025762	Non-glycosylated
579	T	-0.58203318	Non-glycosylated
**585**	T	**0.12893941**	**Potential Glycosylated**
611	T	-0.71182864	Non-glycosylated
616	T	-0.48336726	Non-glycosylated
617	S	-0.61032563	Non-glycosylated
630	T	-0.099202666	Non-glycosylated
631	T	-0.26189087	Non-glycosylated
**634**	S	**0.38903457**	**Potential Glycosylated**
648	S	-0.7462816	Non-glycosylated
**650**	T	**0.14540609**	**Potential Glycosylated**
659	S	-0.37587093	Non-glycosylated
670	S	-1.2718789	Non-glycosylated
672	T	-0.75871608	Non-glycosylated
702	S	-0.51345603	Non-glycosylated
703	T	-0.41902316	Non-glycosylated
708	T	-0.3215481	Non-glycosylated
710	S	-0.04547209	Non-glycosylated
712	T	-0.41708239	Non-glycosylated
729	S	-0.23787628	Non-glycosylated
737	T	-0.11675961	Non-glycosylated
**741**	S	**0.34342161**	**Potential Glycosylated**
744	S	-0.16767934	Non-glycosylated

760	T	-0.55537491	Non-glycosylated
767	S	-0.36375238	Non-glycosylated
770	S	-0.20818358	Non-glycosylated
781	S	-0.27007687	Non-glycosylated
784	T	-0.8063506	Non-glycosylated
**786**	S	**0.31265871**	**Potential Glycosylated**
**794**	T	**0.17128103**	**Potential Glycosylated**
**796**	S	**0.39238508**	**Potential Glycosylated**
797	S	-0.71416416	Non-glycosylated
800	T	-0.85105987	Non-glycosylated
**802**	S	**0.013431216**	**Potential Glycosylated**
**805**	S	**0.15000538**	**Potential Glycosylated**
**819**	S	**0.20165 Potential Glycosylated**	
826	T	-0.53080184	Non-glycosylated
834	S	-0.23857753	Non-glycosylated
843	S	-0.52766058	Non-glycosylated
846	S	-0.10579236	Non-glycosylated
858	T	-0.78701638	Non-glycosylated
859	S	-0.52115287	Non-glycosylated
868	S	-0.77371221	Non-glycosylated
870	T	-0.44949952	Non-glycosylated
882	T	-0.38479173	Non-glycosylated
**887**	T	**0.4844992**	**Potential Glycosylated**
888	T	-0.77111605	Non-glycosylated
889	S	-0.034109026	Non-glycosylated
**892**	S	**1.0262277**	**Potential Glycosylated**
896	T	-0.97943613	Non-glycosylated
900	T	-0.59246102	Non-glycosylated

903	T	-1.0952394	Non-glycosylated
**905**	T	**0.1469529**	**Potential Glycosylated**
**908**	S	**0.92236962**	**Potential Glycosylated**
**911**	S	**0.38860342**	**Potential Glycosylated**
933	S	-0.45179338	Non-glycosylated
**936**	S	**0.0087686924**	**Potential Glycosylated**
947	T	-0.11393568	Non-glycosylated
952	T	-0.35951922	Non-glycosylated
955	T	-0.28395697	Non-glycosylated
958	T	-0.84585122	Non-glycosylated
**960**	T	**1.266187**	**Potential Glycosylated**
**963**	S	**0.1906779**	**Potential Glycosylated**
964	S	-0.16214528	Non-glycosylated
974	T	-0.12857094	Non-glycosylated
**978**	T	**0.035185641**	**Potential Glycosylated**
984	S	-0.54537152	Non-glycosylated
**990**	S	**0.19914269**	**Potential Glycosylated**
**995**	S	**0.39160055**	**Potential Glycosylated**
1022	T	-0.94645925	Non-glycosylated
1033	T	-0.20229347	Non-glycosylated
1039	S	-0.88750058	Non-glycosylated
1045	T	-0.68299768	Non-glycosylated
1046	T	-0.59663598	Non-glycosylated
1055	T	-0.31754778	Non-glycosylated
**1057**	S	**0.19706385**	**Potential Glycosylated**
1058	T	-0.28159235	Non-glycosylated

****
